# Association Between Febuxostat Use and the Incidence of Cardiovascular Events, Mortality, and Kidney Events in Patients With Chronic Kidney Disease Compared to Allopurinol: A Study Using a Japanese Nationwide Database

**DOI:** 10.7759/cureus.70351

**Published:** 2024-09-27

**Authors:** Miho Otani, Yuta Nonomiya, Yasutaka Ihara, Ryota Kawai, Satsuki Taniuchi, Hisako Yoshida, Kazuhiko Tsuruya, Ayumi Shintani

**Affiliations:** 1 Medical Statistics, Osaka Metropolitan University, Osaka, JPN; 2 Nephrology, Nara Medical University, Kashihara, JPN

**Keywords:** cardiovascular events, chronic kidney disease, database, hyperuricemia, kidney prognosis, kidney replacement therapy, mortality, renal prognosis, urate-lowering agent, uric acid

## Abstract

Background

Patients with chronic kidney disease (CKD) were excluded in most trials that investigated the effects of urate-lowering agents, such as febuxostat and allopurinol, in hyperuricemic patients. This exclusion leads to uncertainty regarding the efficacy of febuxostat in patients with CKD. Due to the high prevalence of hyperuricemia in patients with CKD, we aimed to assess the effect of febuxostat on improving patient outcomes concerning cardiovascular events and survival compared with those treated with allopurinol among patients with CKD.

Methods

We conducted a retrospective cohort study using Japanese nationwide administrative data from Jan 1, 2013, to Sep 30, 2020. Patients aged over 60 years diagnosed with CKD were included in this study if they were prescribed either febuxostat or allopurinol. The primary outcome was the occurrence of cardiovascular events including myocardial infarction, stroke, unstable angina requiring urgent revascularization, and all-cause deaths. We estimated hazard ratios (HR) and 95% CI using a Cox proportional hazard regression model adjusted for comorbidities, medications, and laboratory data. We also assessed defined starting kidney replacement therapy as a secondary endpoint treating death as a competing risk using the Fine & Gray regression model.

Results

A total of 21,015 patients included those with febuxostat (n=17,796) and those with allopurinol (n=3,219). The association between the type of drug and the occurrence of cardiovascular events did not show a significant difference (0.107 vs. 0.116 events per patient-year; adjusted HR 0.953, 95% CI: 0.854 to 1.062, P=0.381). Similar results were seen for all-cause deaths (0.060 vs. 0.068 events per patient-year; adjusted HR 0.877, 95% CI: 0.760 to 1.012, P=0.073). Regarding the secondary endpoint, the association between the type of drug and the timing of starting kidney replacement therapy did not show a significant difference (0.118 vs. 0.097 events per patient-year; adjusted HR 0.953, 95% CI: 0.854 to 1.062, P=0.425).

Conclusion

The use of febuxostat was neither associated with a decreased risk of cardiovascular events or deaths nor with the timing of starting kidney replacement therapy compared to the use of allopurinol in patients with CKD.

## Introduction

Hyperuricemia is a common occurrence in patients with chronic kidney disease (CKD), often attributed to diminished uric acid excretion resulting from worsening kidney function. Addressing hyperuricemia in patients with CKD prompts consideration of two critical outcomes: mortality and kidney prognosis. This discussion begins with an exploration of the effects of urate-lowering agents on cardiovascular events and mortality.

In 2018, the CARES trial findings suggested higher all-cause and cardiovascular disease-related mortality in patients with gout and cardiovascular diseases treated with febuxostat compared to those treated with allopurinol [1]. This trial had two shortcomings: the short follow-up period and high dropout rates among patients treated with both febuxostat and allopurinol. Subsequently, the FAST trial reported that febuxostat demonstrated non-inferiority to allopurinol in preventing cardiovascular events and showed no increase in deaths or severe adverse effects with its long-term use [2]. It is important to note that both trials excluded individuals with severe CKD (those whose estimated glomerular filtration rate (eGFR) was less than 30 ml/min/1.73m², i.e., CKD stages 4 and 5).

In another trial concerning urate-lowering agents, the FREED trial reported a lower incidence of kidney impairment, defined as the development of albuminuria or proteinuria or a doubling of serum creatinine levels leading to end-stage kidney disease, in those treated with febuxostat compared to those not treated with febuxostat. In this trial, febuxostat did not significantly impact the serial change in eGFR [3].

In Japan, physicians tend to prescribe urate-lowering agents even for patients with CKD whose eGFR is less than 30 ml/min/1.73 m² [4]. Moreover, it can take several years to more than 10 years for patients with an eGFR of 30 ml/min/1.73 m² to start kidney replacement therapy [5]. Additionally, in this previous study, due to being an open-label trial, there may have been a bias toward patients assigned to the new drug, febuxostat.

Considering the findings of these previous trials, the primary objective of this study is to evaluate the impact of febuxostat, compared to allopurinol, on cardiovascular events, overall survival, and kidney outcomes in patients with CKD, through a comprehensive analysis of real-world data.

Part of this article was previously presented as a poster at the American Society of Nephrology annual meeting on November 5, 2023.

## Materials and methods

Data sources

We used the Diagnosis Procedure Combination (DPC) data in Japan provided by Medical Data Vision (MDV, Tokyo, Japan) [6]. The DPC system is a case-mix patient classification system originally developed in Japan in 2002. MDV’s database includes 44 million people and 470 advanced medical facilities among 1,757 hospitals in Japan. We can access data on prescribed drugs, diagnosed disease names, and procedures such as operations and examinations through records of medical practice and discharge summaries. The large-scale data accumulated by DPC are utilized in clinical research, and numerous studies have used this database [7].

Study population

Inclusion Criteria

We included patients who had their first prescription of either allopurinol or febuxostat from Jan 1, 2013, to Sep 30, 2020. For each subject, the date of their first prescription was designated as the index date. Patients included met all the following criteria: (1) they had been diagnosed with CKD-related disease names (ICD-10 codes listed below) in the DPC system within 18 months before their index date; (2) they had not been prescribed any urate-lowering agents within six months before their index date; (3) they were 60 years or older at their index date. ICD-10 codes related to CKD included chronic nephritis (N030, N032-4, N036, N037, N039, N050-9), CKD (N181-N185, N189, N19), diabetic nephropathy (E102, E112, E132, E142), renal sclerosis (I120, I139), and chronic interstitial nephritis (N119) [8].

Exclusion Criteria

Patients were excluded if they were on dialysis therapy on the index date, had a history of non-fatal myocardial infarction, non-fatal stroke, or coronary artery treatment within the past six months, had a history of congestive heart failure before the index date, were diagnosed with malignant cancer within the past five years [9], did not undergo laboratory exams for serum creatinine within the past three months, or did not have a medical record within the past year of the index date. The ICD-10 codes used for the diagnosis of dialysis therapy and other diseases are provided in Appendix 1.

Outcomes

The primary endpoint was a composite outcome as follows: the first occurrence of all-cause death or cardiovascular diseases including non-fatal myocardial infarction, non-fatal stroke, and unstable angina requiring urgent revascularization. No competing risk was considered, and the day of the last record available in the database was defined as the censoring date for each subject. The secondary endpoint was starting kidney replacement therapy (hemodialysis, peritoneal dialysis, or kidney transplantation) with procedure codes provided in Appendix 1. In the analysis of the secondary endpoint, all-cause deaths were treated as a competing risk, and the day of the last records available in the database was considered the censoring date.

Procedures of patient follow-up

For each subject, the date of their first prescription of either febuxostat or allopurinol was designated as the index date. We followed patients from the index date until the first occurrence of the outcomes or the day of the last record available in the database in the primary and secondary analyses.

Statistical analysis

Baseline clinical characteristics were described using frequencies and proportions for categorical variables, and median and IQR for continuous variables.

For missing values of eGFR, serum uric acid, and hemoglobin, we conducted multiple imputation, in which we generated five imputed datasets using predictive mean matching and integrated the analysis of each dataset.

To assess the time to the first occurrence of the primary endpoint events in patients treated with febuxostat, compared with those treated with allopurinol, we used time-to-event analysis. Data for the patients were censored on the day of the last record in the database. Time-to-event curves were obtained with Kaplan-Meier analyses. The adjusted HRs were obtained with Cox proportional-hazards regression.

The Fine and Gray regression model was used to analyze the time to the first occurrence of the secondary endpoint event considering death as a competing risk. Patients were censored on the day of the last record in the database.

All analyses were adjusted for baseline variables, such as comorbidities (diabetes mellitus, hypertension, dyslipidemia, myocardial infarction, stroke, heart failure, bronchial asthma, chronic obstructive pulmonary disease, peripheral artery disease, cardiac arrest, atrial fibrillation, valve heart disease, liver disease, rheumatoid disease, psychiatric disease, bone fracture, amputation, coronary artery intervention), medications (aspirin, renin-angiotensin system (RAS) inhibitors, diuretics, sodium-glucose transporter 2 inhibitors, statins, ezetimibe, antiplatelets, direct oral anticoagulants, nonsteroidal anti-inflammatory drugs (NSAIDs), calcium channel blockers, insulin, beta blockers, oral anti-diabetic agents, erythropoiesis-stimulating agents, charcoal absorbents, sodium bicarbonate, potassium absorbents, phosphorus absorbents, vitamin D, iron, beta2 agonists, corticosteroid inhalers), laboratory data (eGFR, serum uric acid, and hemoglobin), hospital scale, calendar month of the index date, and department of nephrology.

For sensitivity analyses, we performed propensity score-matched analyses without replacement. Propensity scores were calculated with logistic regression, where the probability of being prescribed febuxostat was the dependent variable as a function of the 49 baseline covariates at the index date (Appendix 2), such as comorbidities (e.g., diabetes mellitus, hypertension), medications (e.g., RAS inhibitor, aspirin, beta blocker), laboratory data (eGFR, serum uric acid, and hemoglobin), hospital scale, and calendar month of the index date. We did 1:1 matching with new users of febuxostat and those of allopurinol by nearest neighbor algorithm with a caliper width of 0.25 SD of the logit of the propensity score. The covariate balance was assessed between patients treated with febuxostat and those treated with allopurinol, with the cutoff of the absolute standardized mean difference of 0.1.

All significance levels were set at a 5% two-sided alpha level. All statistical analyses were conducted using R version 4.1.3 software.

Ethics approval

The Japanese Guidelines for Medical and Biological Research Involving Human Subjects do not apply to clinical studies that use only anonymized data. Informed consent was waived because the data used in this research were anonymized by MDV. We have obtained permission from MDV for third-party disclosure (Appendix 3).

## Results

Study population

**Figure 1:**
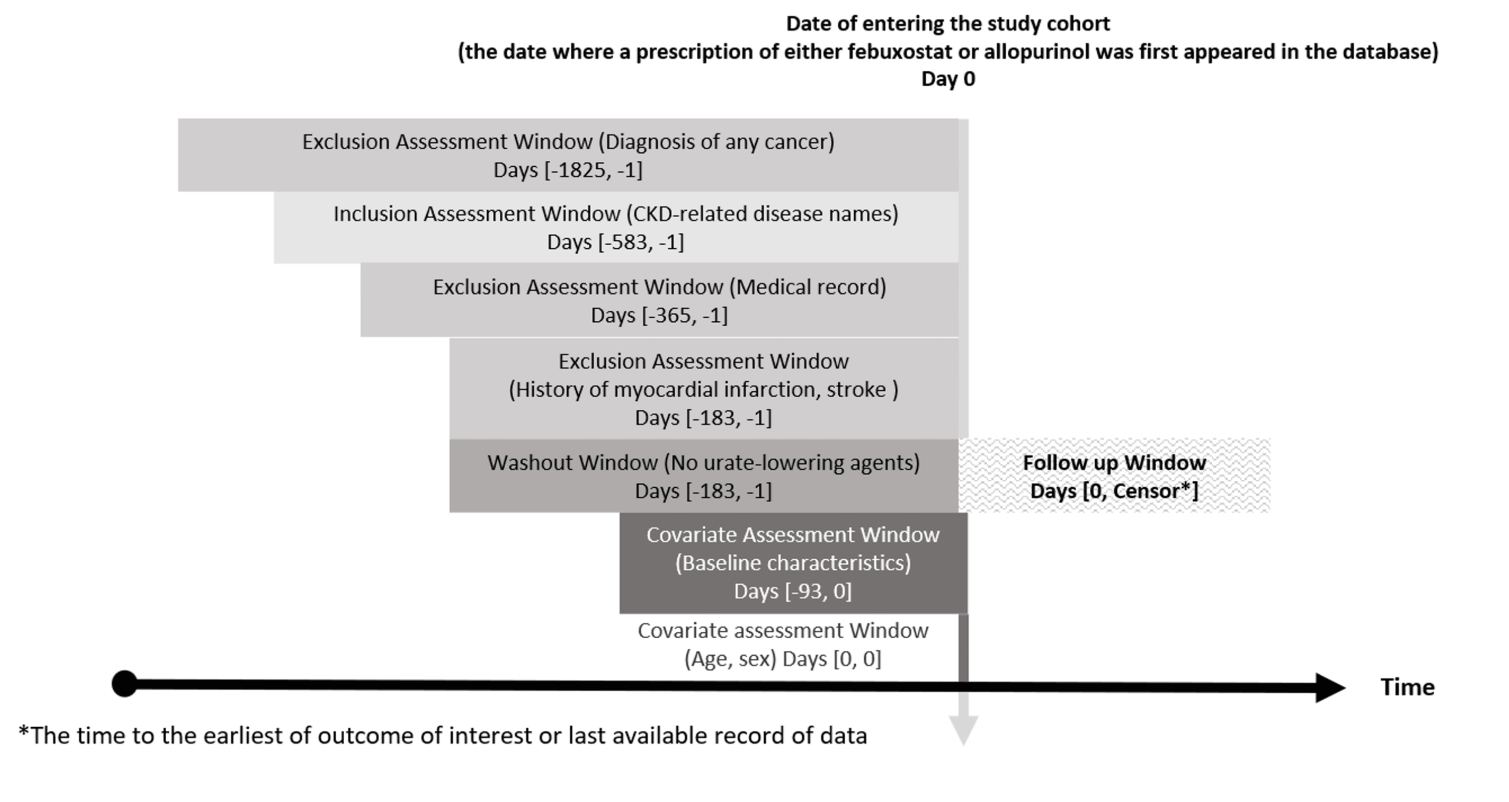
Patient diagram of our study, where cohort entry is based on exposure.

**Figure 2:**
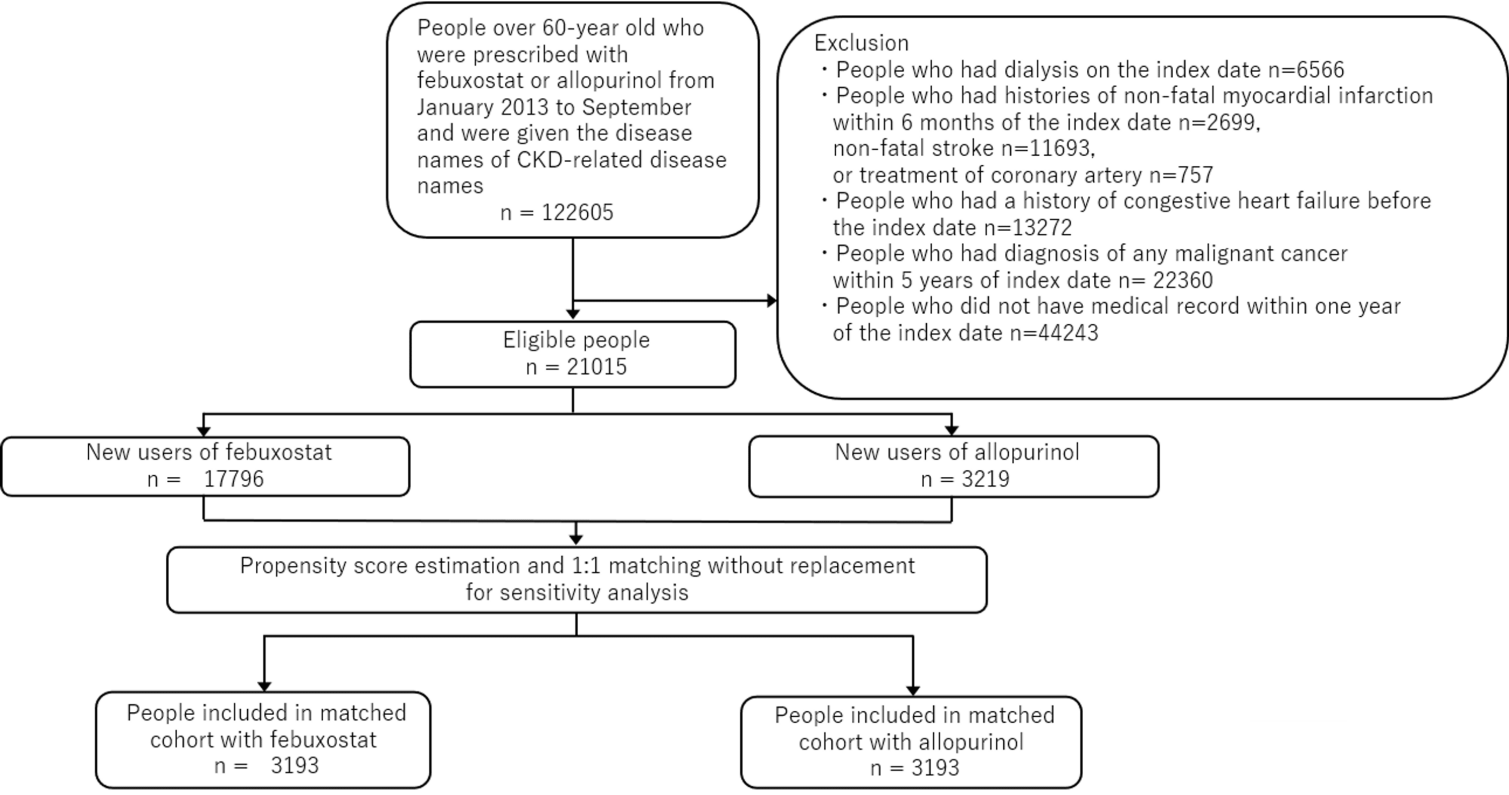
Flowchart of patient inclusion in the study cohort of new users of febuxostat and allopurinol in Japan, from January 2013 to November 2020.

A total of 21,015 patients with CKD were included; of these, 3,219 were allopurinol users and 17,796 were febuxostat users (Figures 1-2). The characteristics of the patients are as follows (Table 1): the number (percentage) of patients with diabetes mellitus was 1,111 (35%) in allopurinol users and 7,593 (42%) in febuxostat users. The medians (IQRs) of age, eGFR, uric acid level, and follow-up duration for allopurinol users versus febuxostat users were 77 (70-84) versus 78 (71-84) years, 28.3 (12.9-42.5) versus 22.5 (13.0-33.9) ml/min/1.73 m², 6.6 (5.3-8.6) versus 8.0 (6.2-9.3) mg/dl, and 16.4 (3.6-34.9) versus 14.6 (4.2-31.5) months, respectively.

**Table 1:** Baseline characteristics of new users of allopurinol and febuxostat. ^1^COPD: Chronic obstructive pulmonary disease; eGFR: Estimated glomerular filtration rate; RAS: Renin-angiotensin system; SGLT2: Sodium-glucose transporter 2; DOACs: Direct oral anticoagulants; NSAIDs: Nonsteroidal anti-inflammatory drugs. Data are presented as n (%) for categorical values or median (interquartile range) for continuous values.

Characteristics^1^	Treatment	
	Allopurinol, N=3,219	Febuxostat, N=17,796
Sex (men, %)	2,163 (67%)	11,277 (63%)
Age (year)	78 (71, 84)	77 (70, 84)
Comorbidity		
Diabetes Mellitus (%)	1,111 (35%)	7,493 (42%)
Hypertension (%)	2,502 (78%)	14,810 (83%)
Dyslipidemia (%)	1,268 (39%)	8,477 (48%)
Myocardial infarction (%)	59 (1.8%)	478 (2.7%)
Stroke (%)	362 (11%)	2,292 (13%)
Heart failure (%)	1,266 (39%)	7,765 (44%)
Bronchial asthma (%)	247 (7.7%)	1,638 (9.2%)
COPD (%)	97 (3.0%)	551 (3.1%)
Peripheral artery disease (%)	249 (7.7%)	1,287 (7.2%)
Cardiac arrest (%)	9 (0.3%)	89 (0.5%)
Atrial fibrillation (%)	514 (16%)	2,867 (16%)
Valve heart disease (%)	768 (24%)	4,219 (24%)
Liver disease (%)	948 (29%)	5,885 (33%)
Rheumatoid disease (%)	203 (6.3%)	1,465 (8.2%)
Psychiatry disease (%)	131 (4.1%)	733 (4.1%)
Bone fracture (%)	483 (15%)	2,302 (13%)
Amputation (%)	2 (<0.1%)	32 (0.2%)
Coronary artery intervention (%)	77 (2.4%)	498 (2.8%)
Laboratory data		
eGFR (ml/min/1.73 m2)	28.3 (12.9, 42.5)	22.5 (13.0, 33.9)
Uric acid (mg/dl)	6.6 (5.3, 8.6)	8.0 (6.2, 9.3)
Hemoglobin (g/dl)	10.7 (9.3, 12.8)	10.9 (9.6, 12.4)
Medication		
Aspirin	236 (7.3%)	1,847 (10%)
RAS inhibitors	528 (16%)	5,735 (32%)
Diuretics	693 (22%)	6,236 (35%)
SGLT2 inhibitors	15 (0.5%)	238 (1.3%)
Statins	301 (9.4%)	3,389 (19%)
Ezetimibe	28 (0.9%)	378 (2.1%)
Antiplatelets	308 (9.6%)	2,161 (12%)
DOACs	115 (3.6%)	766 (4.3%)
NSAIDs	509 (16%)	1,956 (11%)
Calcium channel blockers	654 (20%)	6,366 (36%)
Insulin	373 (12%)	2,725 (15%)
Beta blockers	327 (10%)	2,711 (15%)
Oral anti-diabetic agents	319 (9.9%)	3,360 (19%)
Erythropoiesis-stimulating agents	422 (13%)	3,819 (21%)
Charcoal absorbents	159 (4.9%)	1,205 (6.8%)
Sodium bicarbonate	239 (7.4%)	1,947 (11%)
Potassium absorbents	291 (9.0%)	2,793 (16%)
Phosphorus absorbents	69 (2.1%)	387 (2.2%)
Vitamin D	63 (2.0%)	641 (3.6%)
Iron	190 (5.9%)	1,497 (8.4%)
Beta2 agonists	109 (3.4%)	576 (3.2%)
Corticosteroid inhalers	36 (1.1%)	364 (2.0%)
Hospital scales		
<200 beds	337 (10%)	1,681 (9.4%)
200-499 beds	1,810 (56%)	10,074 (57%)
≥500 beds	1,072 (33%)	6,041 (34%)
Department of nephrology	301 (9.4%)	2,981 (17%)
Index date (months)	52 (32, 71)	62 (43, 77)

Primary and secondary outcomes

**Figure 3:**
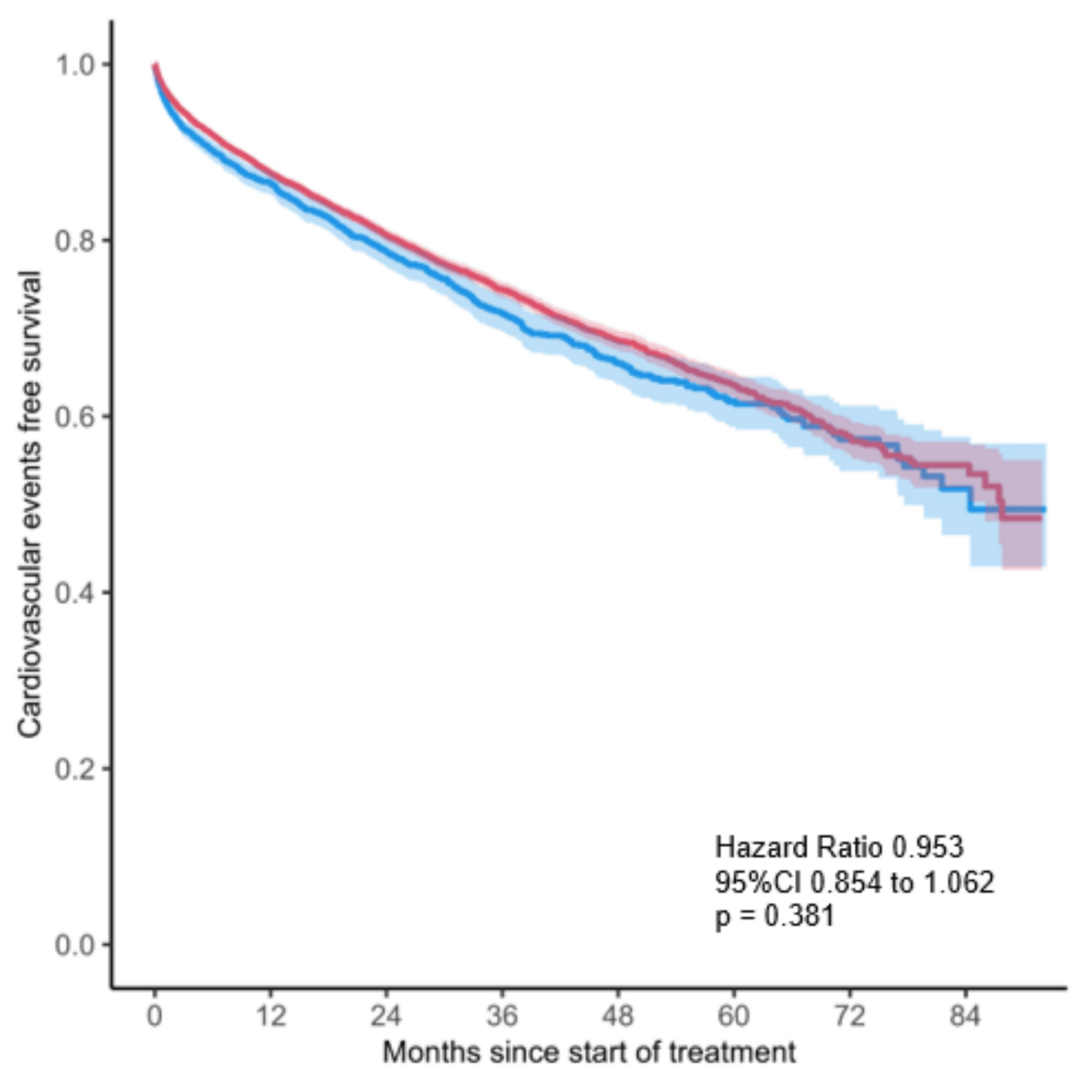
Kaplan-Meier curve of primary endpoints. The cardiovascular event-free survival rate (nonfatal myocardial infarction, stroke, or urgent revascularization due to unstable angina) in users of febuxostat (red) and allopurinol (blue).

The Kaplan-Meier curves for the primary outcome are illustrated in Figure 3. Compared with patients treated with allopurinol, those treated with febuxostat did not show a decreased risk of a composite outcome of cardiovascular diseases and deaths (0.107 versus 0.116 events per person-years in the febuxostat versus allopurinol groups, respectively; adjusted HR 0.953, 95% CI: 0.854 to 1.062, P=0.381).

**Figure 4:**
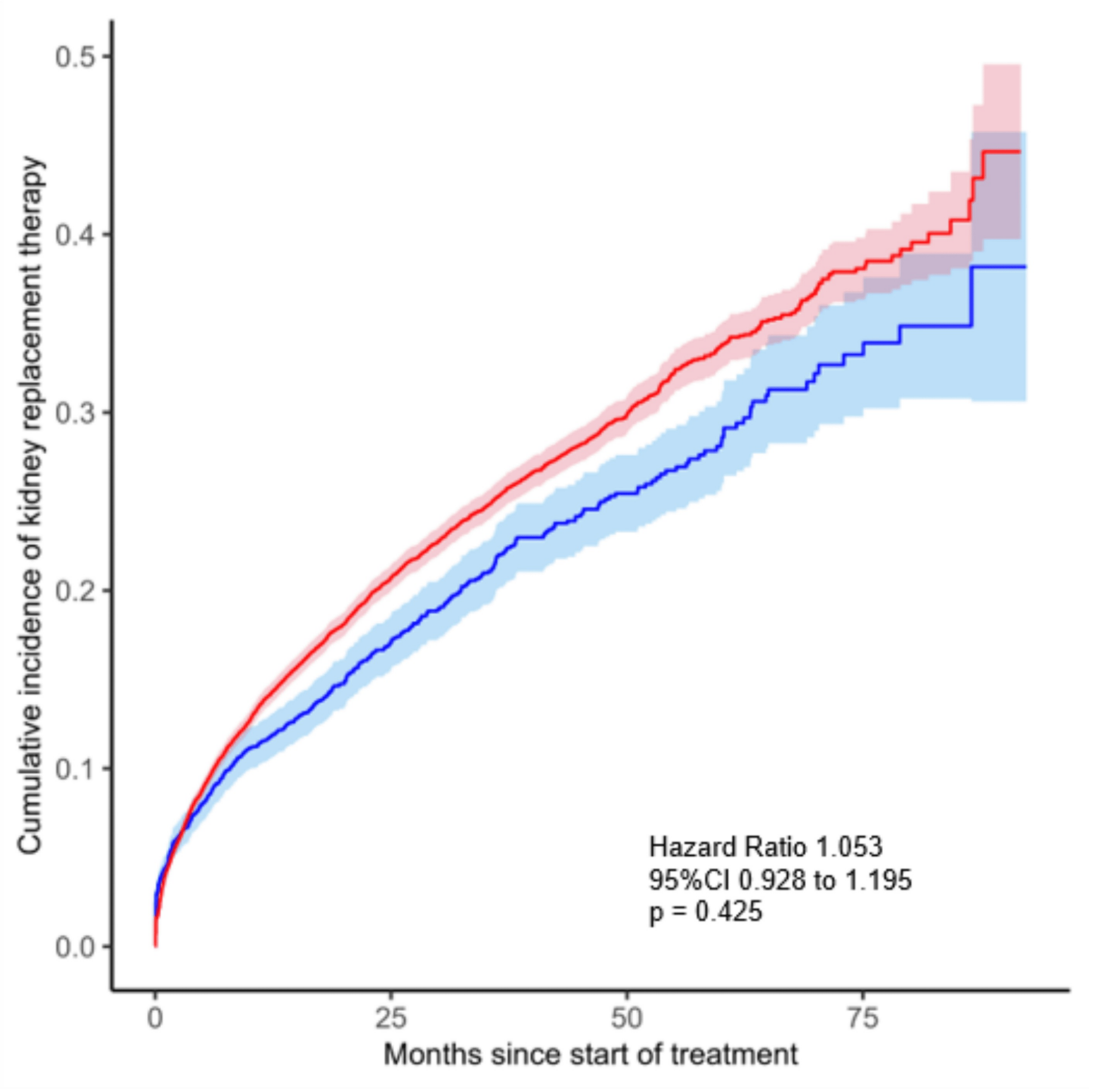
Cumulative incidence of secondary endpoint of starting kidney replacement therapy. The cumulative incidence rate of starting kidney replacement therapy in users of febuxostat (red) and allopurinol (blue).

Secondary endpoint analyses revealed that the use of febuxostat, compared with allopurinol, did not show a decreased risk of starting kidney replacement therapy (0.118 versus 0.097 events per person-years in the febuxostat versus allopurinol groups, respectively; adjusted HR 1.053, 95% CI: 0.928 to 1.195, P=0.425, Figure 4).

Subgroup analyses

**Figure 5:**
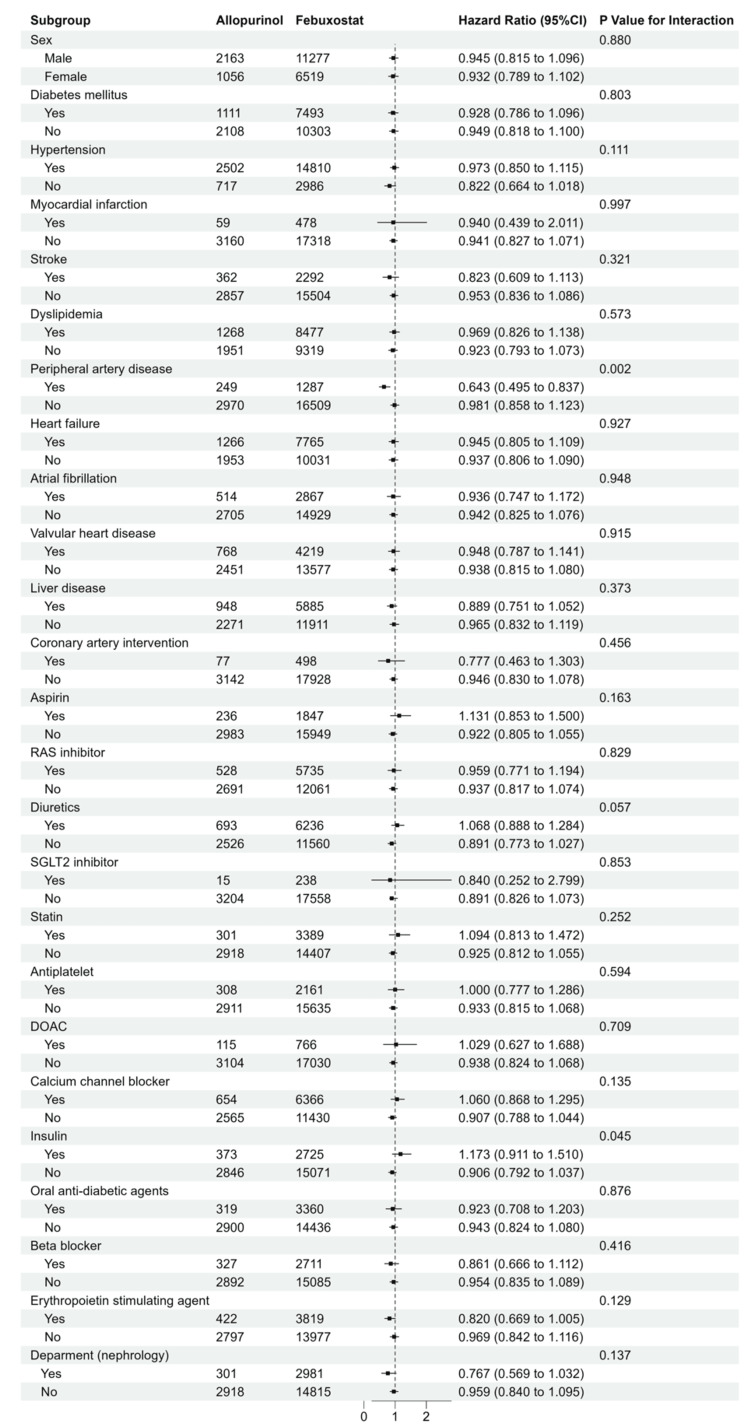
Subgroup analyses of cardiovascular events and deaths with categorical variables. Forest plot for subgroup analyses in users of febuxostat (red) and allopurinol (green).

**Figure 6:**
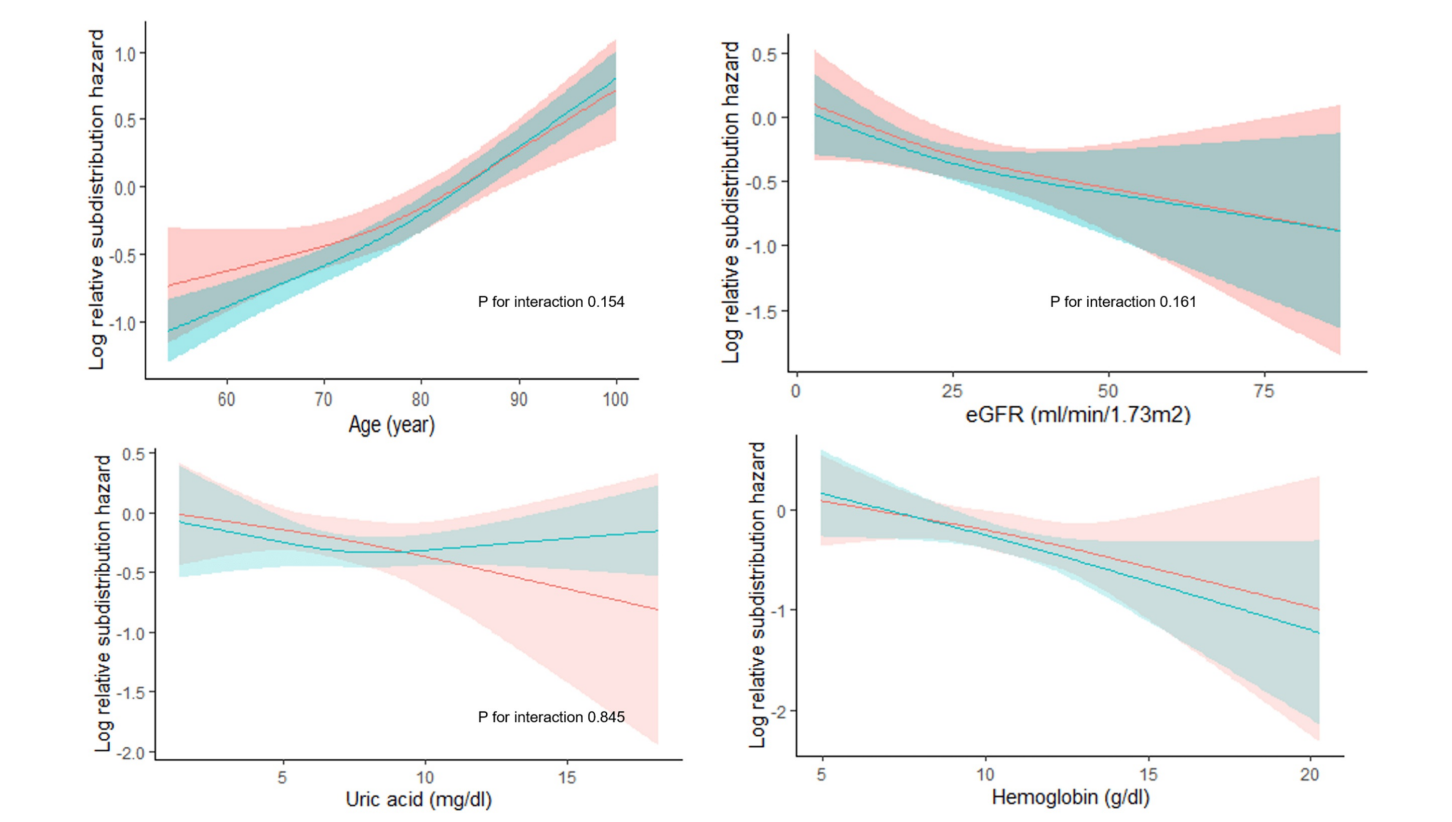
Subgroup analyses of cardiovascular events and deaths with continuous variables. Log relative sub distribution hazard curves in users of febuxostat (red) and allopurinol (green).

In a subgroup analysis considering the primary endpoint, an interaction was observed regarding a history of peripheral artery disease and a prescription of insulin (Figures 5-6). An association with a reduction in the risk of the primary endpoint was found with febuxostat compared with allopurinol in the subgroups with a history of peripheral artery disease (HR: 0.643, 95% CI: 0.495 to 0.837 versus HR: 0.981, 95% CI: 0.858 to 1.123, P for interaction=0.002) and without a prescription of insulin (HR: 0.906, 95% CI: 0.792 to 1.510 versus HR: 1.173, 95% CI: 0.911 to 1.510, P for interaction=0.045). No significant interactions were observed between the use of febuxostat and subgroups based on sex, age, eGFR, and uric acid.

**Figure 7:**
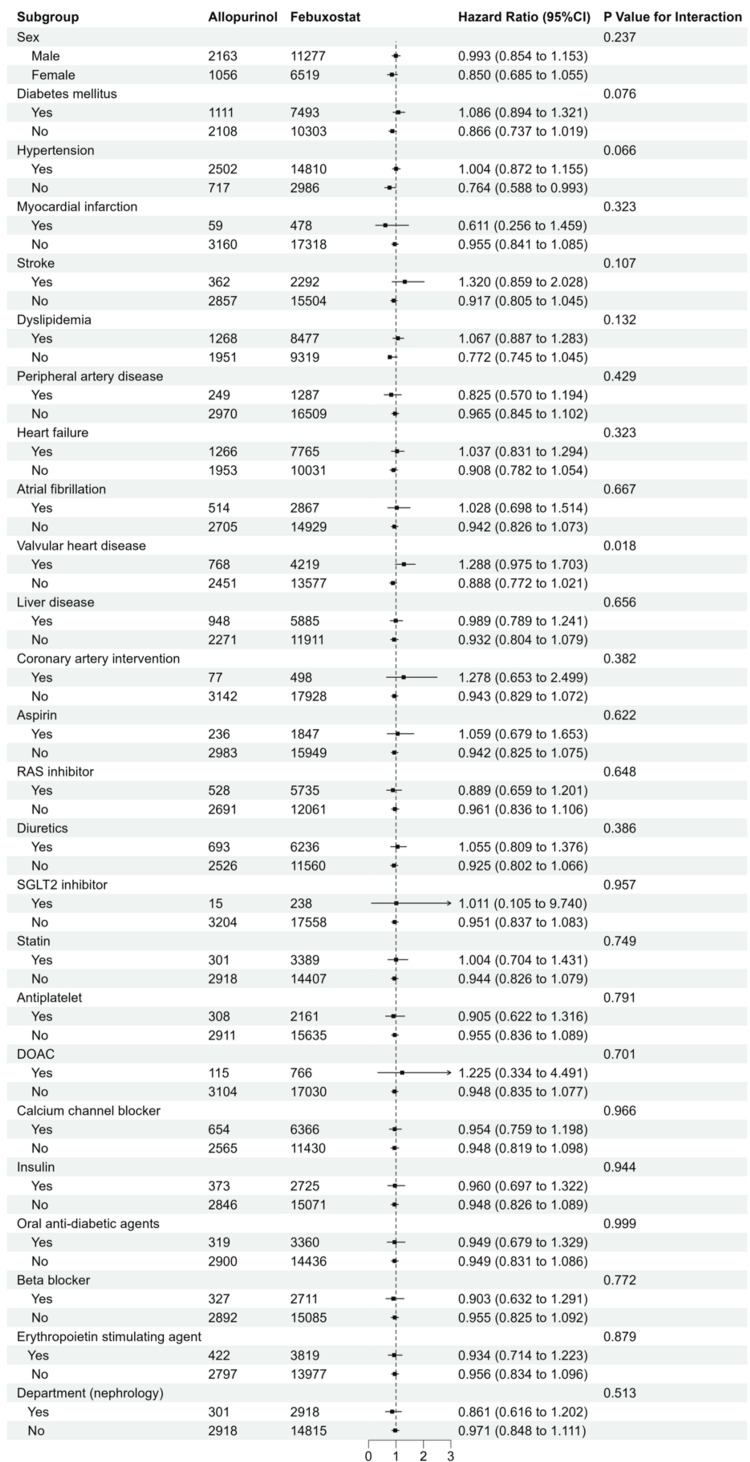
Subgroup analyses of kidney replacement therapy events with categorical variables. Forest plot for subgroup analyses in users of febuxostat (red) and allopurinol (green).

**Figure 8:**
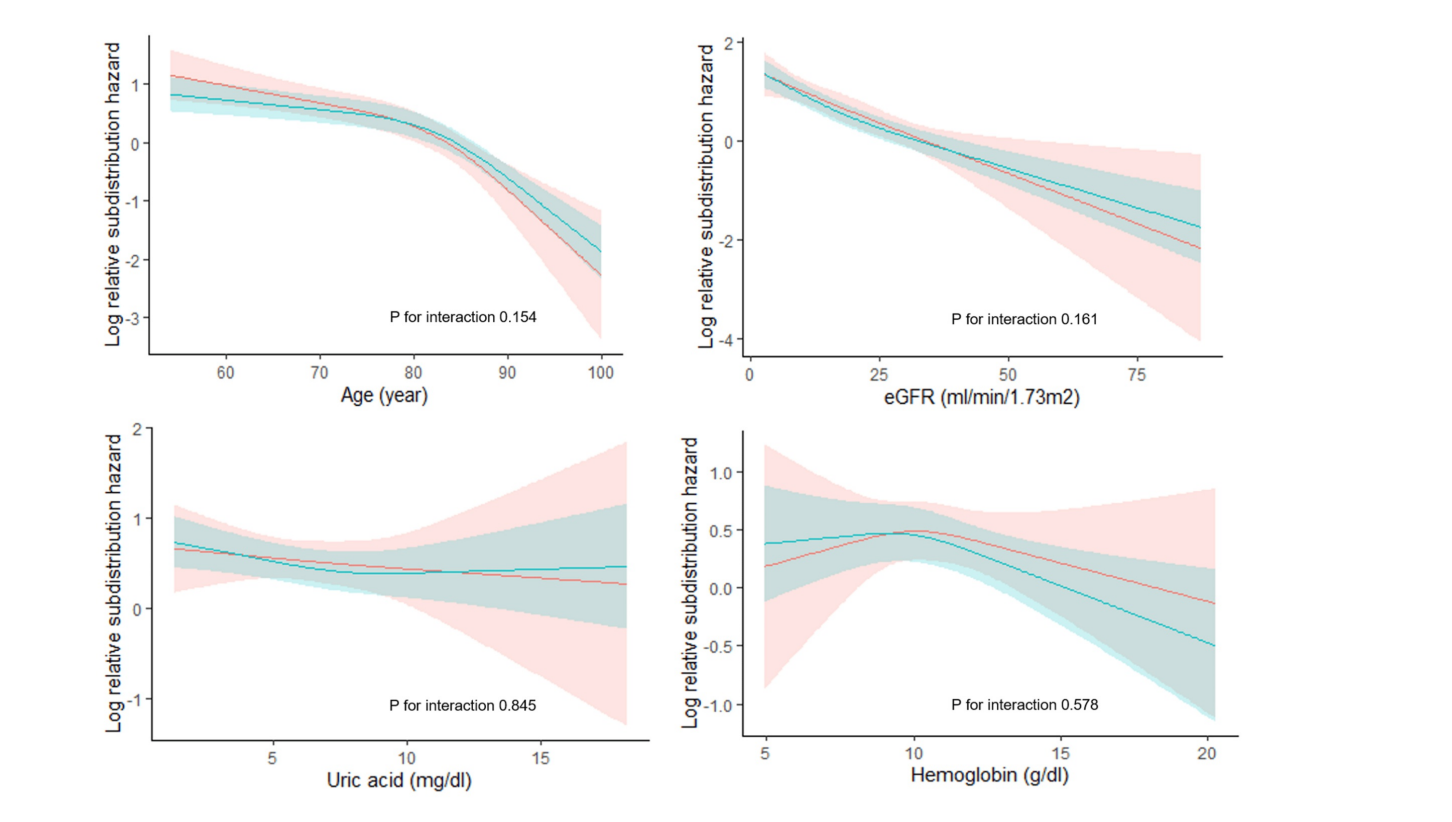
Subgroup analyses of kidney replacement therapy events with continuous variables. Log-relative sub-distribution hazard curves in users of febuxostat (red) and allopurinol (green).

For the secondary endpoint, there was an interaction concerning a history of valvular heart disease (Figures 7-8). An association with a reduction in the risk of starting kidney replacement therapy was observed with the use of febuxostat compared with allopurinol in the subgroup without a history of valvular heart disease (HR: 0.888, 95% CI: 0.772 to 1.021 versus HR: 1.288, 95% CI: 0.975-1.703, P for interaction=0.018).

Sensitivity analyses

Following 1:1 matching without replacement based on the logit of the propensity score, 3,193 new users of allopurinol and 3,193 new users of febuxostat were included. The two groups demonstrated comparable balance in covariates (Appendix 4), although the absolute standardized mean difference for serum uric acid was 0.30, and that for department affiliation (whether the doctor who prescribed the drugs belonged to the department of nephrology or not) was 0.16. The median (IQR) age in both groups was 78 (71-84), with men constituting 2,146 (67.2%) of allopurinol users and 2,201 (68.9%) of febuxostat users. The number of individuals with diabetes mellitus was 1,105 (34.6%) in allopurinol users and 965 (30.2%) in febuxostat users. The medians (IQRs) of eGFR, serum uric acid levels, and follow-up duration were 28.3 (12.9-42.6) versus 27.0 (16.0-41.3) ml/min/1.73 m², 6.6 (5.4-8.6) versus 6.0 (4.6-7.9) mg/dl, and 16.3 (3.6-34.7) versus 17.4 months (4.9-37.1) for allopurinol users versus febuxostat users, respectively.

Primary Outcomes

In comparison with patients treated with allopurinol, those treated with febuxostat did not show an association with a decreased risk of a composite outcome of cardiovascular diseases and deaths (HR: 1.051, 95% CI: 0.952-1.173, P=0.315).

Secondary Outcomes

In comparison with patients treated with allopurinol, those treated with febuxostat did not show an association with the risk of starting kidney replacement therapy (HR: 0.981, 95% CI: 0.870-1.101, P=0.700).

## Discussion

In the present study, patients treated with febuxostat did not show an association with the primary endpoint, designated as a composite outcome of cardiovascular events and all-cause deaths, nor with the secondary endpoint, designated as starting kidney replacement therapy, compared to those treated with allopurinol. These findings remained robust when employing the propensity score matching method.

Several large clinical trials have explored the impact of febuxostat compared to allopurinol on cardiovascular events and mortality. Our study contributes valuable insights into the use of febuxostat in patients with CKD, utilizing data from a Japanese nationwide database. This database contained 44 million patients and included 860,000 patients with CKD, resulting in about 21,000 patients with CKD who were new users of febuxostat or allopurinol. To eliminate immortal time bias, we used a new user design, including only patients who had not received a prescription for any urate-lowering agents within 6 months before their initial prescriptions of the targeted drugs. To remove selection bias, we performed multiple imputations for missing data. To minimize the risk of confounding, we used the propensity score matching method with many characteristics of patients, including the information of year-month at the index date for sensitivity analysis. Notably, the kidney function of our patients was severely compromised, with a median eGFR of 28 ml/min/1.73 m².

Regarding the primary endpoint, patients treated with febuxostat, compared to those treated with allopurinol, were not associated with a reduction of cardiovascular events and deaths. The results were similar in a sensitivity analysis. This result is consistent with the findings of the FAST trial. The median eGFR of the population in our study was 28 ml/min/1.73 m², thus we showed the result among patients with severe CKD. Moreover, in the subgroup analysis, we demonstrated no significant difference in cardiovascular events and deaths between febuxostat and allopurinol among patients whose eGFR was less than 30 ml/min/1.73 m². Since no clinical trials comparing the effects of febuxostat and allopurinol on the risk of cardiovascular events and deaths have been conducted in patients with severe CKD, this is the first report that demonstrated no significant association between cardiovascular events and deaths and febuxostat in patients with severe CKD.

Regarding kidney prognosis as well, our study found that patients treated with febuxostat were not associated with a reduction in the initiation of kidney replacement therapy compared to patients treated with allopurinol. The FREED trial showed that febuxostat improved kidney prognosis compared to allopurinol, whereas our result contrasts with their findings. Our subgroup analyses revealed that there was no interaction between febuxostat and kidney prognosis compared with allopurinol among patients with an eGFR of 30 ml/min/1.73 m² or higher, a similar population regarding kidney function to those in the FREED trial. The difference in the severity of outcomes may account for the disparity in results; our outcome focused on kidney replacement therapy, whereas that of the FREED trial was on the development of microalbuminuria, mild proteinuria, progression to overt albuminuria, doubling of serum creatinine level, and progression to end-stage kidney disease. In fact, the FREED trial showed that febuxostat did not improve the serial change of eGFR compared to allopurinol. Furthermore, the fact that allopurinol was administered to only 27.2% of the patients in the control group of the FREED trial would significantly contribute to the differences observed in these results. We did not compare hyperuricemic patients treated with febuxostat to those untreated in this research, while the FEATHER trial reported that the use of febuxostat did not improve kidney prognosis in CKD stage 3 patients with asymptomatic hyperuricemia compared to untreated placebo, suggesting that febuxostat may not provide kidney protection [10]. One reason for the lack of significant findings might be the shortness of the follow-up period. The median follow-up period was 16.3 months for patients prescribed with allopurinol and 17.4 months for those prescribed with febuxostat.

The present study has several limitations. First, this is a database study, and the definition of exposure relies solely on a history of prescription. Therefore, details regarding drug adherence are unavailable, potentially impacting study outcomes if drug adherence is low. Since this study compares the two drugs, if the levels of drug adherence are similar in both groups, the results would likely be less susceptible to this issue. Second, our study did not account for variations in drug dosage. Allopurinol dose is typically adjusted based on the patient’s kidney function, while febuxostat dose is recommended at 10 mg/day initially for patients with eGFR less than 50 ml/min/1.73 m², with the potential for dose escalation. Therefore, the impact is expected to be minimal because the doses of both drugs are designated well. Third, the lack of data on urine examination in the utilized database precludes consideration of urine-related factors, despite reports linking proteinuria to an increased risk of cardiovascular diseases [11] and kidney replacement therapy [12]. Fourth, limited information on hospital transfers in the utilized database of the current study hinders a comprehensive understanding of events such as death, cardiovascular events, and initiation of kidney replacement therapy. Nevertheless, considering that this happens similarly in both groups, this is not a significant bias in this research. The use of the Japanese nationwide database raises questions about the generalizability of our results to other country populations.

## Conclusions

Our research indicates that the use of febuxostat was neither associated with a decreased risk of cardiovascular events or deaths nor with the timing of starting kidney replacement therapy compared to the use of allopurinol in patients with CKD. It is still unclear how we should manage hyperuricemia in patients with CKD, with some researchers considering hyperuricemia to be merely a consequence of CKD. Further research is required to determine the optimal management of hyperuricemia in CKD.

## Figures and Tables

**Figure 9:**
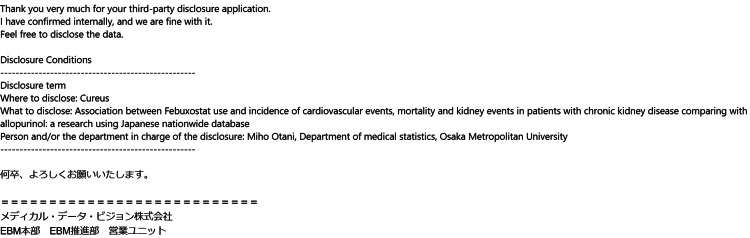
Obtained permission from MDV for third-party disclosure. MDV: Medical Data Vision.

## Appendices

Appendix 1

**Table 2:** List of ICD-10 codes and receipt codes for diseases and medical procedures.

Disease/procedure	Procedure codes icd10 code/receiptcode
Chronic kidney disease	(icd10 code) N030, 32-4, 36, 37, 39, N050-9, N181-N185, N189, N19, E102, E112, E132, E142, I120, I139, N119
Urate-lowering agents	(receiptcode) 610463076, 610463077, 620003074, 620003511, 620003513, 620003514, 620003639, 620004446, 620004449, 620004496, 620004574, 620004921, 620005059, 620005115, 620005961, 620006825, 620007142, 620007143, 620856201, 620856206, 620856208, 620856209, 620856210, 620856211, 620856213, 620856215, 620856217, 620856218, 620856223, 620856230, 620856241, 620856242, 620856243, 620856402, 620856601, 622001501, 622002402, 622005002, 622006202, 622009301, 622009302, 622009502, 622013302, 622018601, 622018602, 622019001, 622019002, 622021701, 622026902, 622031801, 622032301, 622032302, 622036102, 622037801, 622038602, 622040301, 622051001, 622051002, 622103102, 622310700, 622310800, 622739700, 613940025, 620005964, 621973601, 622265801, 622265901, 622266001, 622266201, 622266301, 622266401, 622703601, 622703701, 622703801, 622057201, 622057301, 622057401, 613940032, 613940056, 620006600, 610431121, 613940047, 613940048, 620002547, 620002548, 620003584, 620003621, 620005052, 620007074, 620009402, 620857007, 620857301, 620857303, 620857304, 620857306, 620857310, 620857312, 620857407, 621907601, 622050702, 622073701, 622080501, 622080502, 622103001, 622103002, 622323700, 622739800, 622739900
Aspirin	(receiptcode) 610443053, 620000065, 620001952, 620002761, 620006661, 620007816, 620009301, 621525202, 621675501, 621676502, 622258001, 610443056, 621374801, 621374901, 621375001, 621419201, 621419401
Renin-angiotensin system inhibitors	(receiptcode) 610422142, 610433007, 610433017, 610433068, 610433069, 612140479, 612140480, 620001878, 620281201, 620281304, 620281601, 620281801, 620282504, 620282601, 621245901, 621987002, 621992502, 622064502, 622309900, 612140711, 612140712, 612140713, 612140711, 612140712, 612140713, 612140711, 612140712, 612140713, 612140711, 612140712, 612140713, 612140711, 612140712, 612140713, 612140711, 612140712, 612140713, 612140711, 612140712, 612140713, 612140711, 612140712, 612140713, 612140711, 612140712, 612140713, 612140711, 612140712, 612140713, 612140711, 612140712, 612140713, 612140711, 612140712, 612140713, 612140711, 612140712, 612140713, 612140711, 612140712, 612140713, 612140711, 612140712, 612140713, 610444028, 610444030, 610444062, 610444064, 610444075, 610444076, 610444119, 610444120, 610444153, 610444160, 610444161, 610444162, 610444163, 610444164, 610444165, 610444166, 610444167, 610444169, 610444170, 610453025, 610463039, 612140443, 612140444, 612140445, 620001876, 620001877, 620002009, 620002010, 620006563, 620006745, 620007943, 620007944, 620008291, 620009306, 620009307, 621243302, 621243401, 621243402, 621243601, 621243701, 621243801, 621244202, 621244402, 621244501, 621244502, 621244602, 621244702, 621245002, 621245201, 621245301, 621245402, 621245501, 621245602, 621245801, 621401303, 621401401, 621401501, 621401603, 621401701, 621573601, 621573701, 621632201, 621632301, 621642802, 621642902, 621890901, 621891001, 621975301, 621975302, 621977601, 621977602, 622023701, 622023901, 622025401, 622025404, 622025504, 622028801, 622031901, 622038702, 622039401, 622064401, 622064403, 622067201, 622067202, 622101901, 622108001, 622388301, 62288401, 622389301, 622689700, 622721700, 610412013, 610412039, 61012040, 610412044, 610412045, 610453036, 610453037, 610461106, 61061108, 612140074, 612140075, 612140489, 620006879, 620008602, 620008603, 621365602, 621365713, 621365736, 621365810, 621365821, 621365822, 621365823, 622312300, 622721600, 610407142, 610407143, 610407144, 612140557, 612140558, 612140559, 620001964, 621629301, 621635101, 621683601, 612140720, 612140721, 612140722, 620009355, 620009357, 620009358, 620009359, 620009360, 620009362, 620009363, 620009365, 620009366, 620009367, 620009368, 620009370, 620009371, 620009372, 620009373, 620009374, 620009375, 620009376, 620009377, 620009378, 620009379, 620009380, 620009381, 620009383, 620009384, 620009385, 620009386, 620009389, 620009391, 620009392, 621945101, 621945201, 621945301, 621963001, 621963201, 612140492, 612140493, 612140494, 610409327, 610409328, 610409331, 610409332, 620000075, 620000076, 620000126, 621526102, 621526202, 621526301, 621530201, 621865301, 621866501, 622722900, 612140704, 612140705, 620000133, 621525601, 621530501, 621684301, 622100302, 610421318, 610421319, 620002718, 620002719, 620003262, 620003263, 621682801, 621682901, 621689701, 621689801, 622723100, 610444017, 610444154, 610444172, 610444178, 612140647, 612140648, 612140649, 612140650, 612140651, 612140652, 620002630, 620006158, 620006746, 620009453, 621246201, 621246301, 621246401, 621246501, 621402202, 621676601, 621945401, 621945501, 621966002, 621966202, 621966302, 622076902, 622077002, 622102201, 622102301, 622324900, 622325000, 622722500, 622143501, 622143601, 622341401, 620006793, 620006794, 620006796, 620006797, 622242401, 622249501, 622577201, 622577301, 622577401, 622579401, 622579501, 622579601, 622579701, 622579801, 622579901, 622582901, 622583001, 622586801, 622586901, 622587001, 622587501, 622587601, 622587701, 622594101, 622594201, , 622594301, 622598701, 622598801, 622598901, 622599601, 622599701, 622601601, 622601701, 622601801, 622601901, 622602001, 622602101, 622633801, 622633901, 622641401, 622641501, 622641601, 622664801, 622664901, 622689500, 622725700, 620001905, 620001906, 620003525, 621982301, 622452801, 622452901, 622454201, 622540001, 622571101, 622571201, 622571301, 622571401, 622574701, 622574801, 622574901, 622575001, 622577601, 622577701, 622577801, 622580001, 622580101, 622580201, 622580301, 622584301, 622584401, 622584501, 622584601, 622585601, 622585701, 622585801, 622585901, 622587801, 622587901, 622588001, 622588101, 622589401, 622589501, 622589601, 622589701, 622591301, 622591401, 622591501, 622591601, 622592101, 622592201, 622592301, 622592401, 622593301, 622593401, 622593501, 622593601, 622593701, 622593801, 622594901, 622595001, 622595101, 622595201, 622597201, 622597601, 622597701, 622597801, 622597901, 622598001, 622598101, 622598201, 622599901, 622600001, 622600101, 622600201, 622600401, 622600501, 622602201, 622602301, 622602401, 622602501, 622602601, 622602701, 622602801, 622602901, 622603001, 622603101, 622603201, 622603301, 622603401, 622603501, 622603601, 622603701, 622620301, 622620401, 622620501, 622631701, 622631801, 622631901, 622652101, 622663701, 622663801, 622663901, 622690200, 622690400, 622690600, 622690800, 610432011, 610432012, 610432013, 610432014, 622357801, 622357901, 622358001, 622358101, 622365401, 622365501, 622365601, 622365701, 622367401, 622367501, 622367601, 622367701, 622368401, 622368501, 622368601, 622368701, 622369201, 622369301, 622369401, 622369501, 622369601, 622369701, 622369801, 622369901, 622370401, 622370501, 622370601, 622370701, 622371301, 622371401, 622371501, 622371601, 622372001, 622372101, 622372201, 622372301, 622373401, 622373501, 622373601, 622373701, 622376501, 622376601, 622376701, 622376801, 622377001, 622377101, 622377201, 622377301, 622377401, 622377501, 622377601, 622377701, 622378401, 622378501, 622378601, 622378701, 622379601, 622379701, 622379801, 622379901, 622380501, 622380601, 622380701, 622380801, 622381501, 622381601, 622381701, 622381801, 622382201, 622382301, 622382401, 622382501, 622382601, 622382701, 622382801, 622382901, 622383701, 622383801, 622383901, 622384001, 622385001, 622385101, 622385201, 622385301, 622385401, 622385501, 622385601, 622386001, 622386401, 622386501, 622386601, 622386701, 622387101, 622387201, 622390201, 622390301, 622390401, 622390501, 622390601, 622390701, 622390801, 622390901, 622391301, 622391401, 622391501, 622391601, 622392601, 622392701, 622392801, 622392901, 622393501, 622393601, 622394201, 622394301, 622395401, 622395501, 622395601, 622395701, 622396101, 622396201, 622396301, 622396401, 622397701, 622397801, 622397901, 622398001, 622398101, 622398201, 622398301, 622398401, 622398501, 622398601, 622398701, 622398801, 622398901, 622399001, 622399101, 622399201, 622399301, 622399401, 622399501, 622399601, 622399701, 622399901, 622400001, 622412101, 622412201, 622412301, 622412401, 622459201, 622459301, 622459401, 622459501, 622474100, 622474200, 622474300, 622474400, 622474500, 620002429, 620002430, 621999101, 622535501, 622535601, 622535701, 622536101, 622536201, 622536301, 622537001, 622537101, 622537201, 622540501, 622540601, 622540701, 622540901, 622541001, 622541101, 622543701, 622543801, 622543901, 622544001, 622544101, 622546001, 622546101, 622546201, 622546501, 622547101, 622547201, 622549301, 622549401, 622549501, 622551501, 622551601, 622551701, 622553401, 622553501, 622553601, 622557601, 622557701, 622557801, 622558101, 622558201, 622558301, 622558401, 622558501, 622559501, 622559601, 622559701, 622562801, 622562901, 622563001, 622563601, 622563701, 622563801, 622563901, 622564001, 622564101, 622565101, 622565201, 622566601, 622566701, 622566801, 622568801, 622568901, 622569001, 622570501, 622570601, 622570701, 622571501, 622571601, 622571701, 622613800, 622613900, 622614000, 622614100, 622636701, 622639701, 622639801, 622691300, 610443042, 610443043, 610443044, 620002422, 622255401, 622255501, 622255601, 622255701, 622307501, 622307601, 622307701, 622307801, 622308701, 622308801, 622308901, 622333601, 622333701, 622333702, 622333801, 622333802, 622333901, 622333902, 622334401, 622334501, 622334901, 622335001, 622335101, 622335201, 622336901, 622337001, 622337101, 622337401, 622337501, 622337601, 622337701, 622338801, 622338901, 622339001, 622339101, 622339201, 622340401, 622340402, 622340501, 622340502, 622340601, 622340602, 622340701, 622340702, 622341001, 622341101, 622341201, 622341301, 622342101, 622342201, 622342301, 622343101, 622343201, 622343301, 622343401, 622343601, 622343701, 622343801, 622343901, 622345701, 622345801, 622345901, 622346001, 622346401, 622346501, 622346601, 622346701, 622347501, 622347601, 622347701, 622348001, 622348101, 622348201, 622348301, 622348801, 622348901, 622349001, 622349101, 622349801, 622349901, 622350001, 622350101, 622350801, 622350901, 622351001, 622351101, 622351601, 622351602, 622351701, 622351702, 622351801, 622351901, 622352501, 622352601, 622352701, 622352801, 622353201, 622353301, 622353401, 622353501, 622353601, 622353701, 622353901, 622354101, 622354201, 622355001, 622355101, 622355201, 622355701, 622355801, 622355901, 622356001, 622356701, 622357101, 622357201, 622357301, 622357401, 622358701, 622358801, 622358901, 622359001, 622359401, 622359501, 622359601, 622359701, 622361601, 622361701, 622361901, 622362001, 622362101, 622362201, 622362301, 622362601, 622451801, 622451901, 622452001, 622452101, 622456501, 622456601, 622456701, 622456801, 622465701, 622465801, 622465901, 622466001, 622469401, 622469501, 622469601, 622469701, 622474600, 622474700, 622474800, 622474900, 622615200, 622615300, 622615400, 610421329, 610421330, 620009106, 620009126, 620009127, 622134301, 622134401, 622134501, 622136701, 622136801, 622136901, 622137801, 622137901, 622138901, 622139001, 622139101, 622140101, 622140201, 622140301, 622142601, 622142701, 622142801, 622144801, 622144901, 622145001, 622145901, 622146001, 622146101, 622146801, 622146901, 622147001, 622150101, 622150201, 622150301, 622151701, 622151801, 622152401, 622152501, 622152601, 622153001, 622153101, 622153201, 622153501, 622153601, 622153701, 622155001, 622155002, 622155101, 622155102, 622156601, 622156701, 622158001, 622158101, 622158201, 622160301, 622160401, 622160501, 622162201, 622162301, 622162401, 622163001, 622163101, 622163201, 622165301, 622165401, 622165501, 622166501, 622166601, 622166701, 622168601, 622168701, 622168801, 622171001, 622171101, 622171201, 622173501, 622173601, 622173701, 622174601, 622174701, 622176001, 622176101, 622177001, 622177101, 622177201, 622181201, 622181301, 622325700, 622473900, 622474000, 622267201, 622267301, 620009090, 620009091, 622495601, 622495701, 622506801, 622506901, 621926601, 621926701, 622535301, 622535401, 622543501, 622543601, 622547501, 622547701, 622558801, 622558901, 622569301, 622569401, 622613600, 622613700, 620009092, 620009093, 622495801, 622495901, 622496601, 622496701, 622513501, 622513601, 622515101, 622520001, 622520101, 622524301, 622524401, 622527001, 622527201, 622527301, 622529101, 622529201, 620004414, 621769401, 621769402, 622279501, 622309101, 622334001, 622334002, 622334701, 622335501, 622337901, 622339401, 622340801, 622342501, 622343501, 622344401, 622346101, 622346801, 622347301, 622348401, 622348601, 622349301, 622351201, 622352401, 622353801, 622354501, 622355301, 622356101, 622356901, 622357001, 622357501, 622358601, 622359801, 622362901, 622363001, 622443001, 622444401, 622454801, 622459101, 622463201, 622464201, 622472701, 622479801, 622480901, 622481801, 622482101, 622483301, 622486501, 622496501, 622499901, 622501601, 622502801, 622504501, 622504901, 622505801, 622507201, 622512601, 622520501, 622617500, 622617600, 622341701, 622341801, 622199201, 622199301, 622620101, 622620201, 622622701, 622622801, 622625201, 622625301, 622625901622626701, 622626801, 622627501, 622627601, 622629301, 622631501, 622631601, 622632001, 622632101, 622632301, 622632401, 622632601, 622632701, 622633601, 622633701, 622635301, 622635401, 622635501, 622635601, 622637501, 622637601, 622642501, 622642601, 622689300, 622689400, 21974501, 621974601, 621986501, 621986601, 622460201, 622460301, 622467301, 622467401, 622468801, 622468901, 622519601, 622519701, 622524101, 622524201, 622543001, 622543101, 622006801, 622224601, 622535101, 622535201, 622543301, 622543401, 622547301, 622547401, 622558601, 622558701, 622566901, 622567001, 622569101, 622569201, 622570301, 622570401, 622593901, 622594001, 622613400, 622613500, 622516502, 621980801, 622422901, 622443101, 622443501, 622443901, 622444501, 622445301, 622446601, 622448001, 622451101, 622451501, 622453401, 622454301, 622455101, 622455501, 622456301, 622457301, 622458401, 622460101, 622463101, 622463801, 622464301, 622465201, 622468201, 622468301, 622468401, 622481001, 622493501, 622501701, 622542801, 622615100, 622314301, 622820901, 622821001, 622821101, 621932501
Diabetes mellitus	(icd10code) E10, E11, E110-6, E117, E119
Hypertension	(icd10code) I10, H350, I110, I120, I674, I151, I159, I119, I129, I139, H350, I152, I619, I150, H208, I270, O11, O141, D350
Nonfatal myocardial infarction	(icd10code) I219, I210-3, I234, I232, I233, I231, I236, I230, I235, I221, I229, I220, I228, I241,
Nonfatal stroke	I633, I639, I638, I635, I693, I634, I636, I630, I631, I632
Coronary angioplasty	(receiptcode) 150374910, 150375010, 150375110, 150260350, 150284310, 150359310, 150443750, 150375210, 150375310, 150375410, 160107550, 150318310, 150145710, 150145810, 150145910, 150146010, 150302770, 150318410, 150318510, 150143110, 150318610, 150318810, 150319110, 150319410, 150318910, 150319210, 150319510, 150328110
Dyslipidemia	(icd10code) E785, N280, K868, E780, E786
Congestive heart failure	(icd10code) I099, I110, I500, I501, I509
Bronchial asthma	(icd10code) J459, J450, I501, J451, J458, O995, J46
COPD	(icd10code) J449, J448, J441
Peripheral artery disease	(icd10code) I739, I7020, I744, I771, I7021
Cardiac arrest	(icd10code) I469, I460
Rheumatoid disease	(icd10code) M0610, M0630, M0620, M0691-9, M0690, M0680-1, 3-7, M0600-9, M0530, M0510, M0520, M0500, M0590-9, M0580-7, M0820, M0890, M0830, M0810, M084, M088, M0800-7, M0845, M0844, M0847, M0840, M0841, M0846, M0843, M0842, M0885, M0884, M0887, M0880, M0825, M0824, M0827, M0820, M0881, M0886, M0883, M0887, M0821, M0826, M0823, M0827, M0882, M0822, M300, M308, M303, M302, M301, M312, M319, M316, M318, M310, M314, M311, M317, M313, M329, M320, M321, M330, M331, M332, M349, M348, M341, M340, M359, M355, M352, M357, M353, M356, M351, M354, M358, M350, M45-0, M45-9
Psychiatry disease	(icd10code) F04, F059, F051, F050, F058, F068, F067, F060, F063, F064, F065, F062, F069, F061, F066, F060, F071F072, F079, F070, F078, F09, F100, F101, F105, F102, F107, F104, F106, F103, F209, F201, F205, F208, F204, F200, F202, F206, F203
Bone fracture	(icd10code) C79, C795, M966, 8, M8435-9, M8442, 5, 8-9, M4849, M8402-6, 8, M841, M8421-3, 6, 9, M8000-1, 3, 6, 8, M8050-3, 6, 8, M8092, 5, M8020-3, 5, 6, 8, M8080-3, 5, 6, 8, M8040-3, 5-6, 8-9, M801-4, 10-13, 15-6, 18, M8030-3, 5, 6, 8, M809, M8090-1, 3, 6, 8, 9 M8696, M8415, P130, 4, S4290-1, S8280-1, S9240-1, S6250-1, S0280-1, S1280-1, , S3220-1, S9210-1, S3280-1, S9270-1, S5290-1, S9290-1, S7220-1, S5280-1, S0240-1, S2220-1, S8210-1, S9200-1, S3230-1, S8270-1, S4210-1, S6260-1, S6270-1, S729, S7290-1, S521, S2230-1, S3240-1, S1270-1, S4200-1, S9250-1, S0230-1, S0210-1, S3210-1, S5270-1, S523, S5230-1, S5240, S0220-1, S2240-1, S1290-1, S6280-1, S1210-1, S3200-1, S0260-1, S1200-1, S2200-1, S7200-1, S5221, S12, S825, S8251, S7270-1, S925, S8240-1, S8200-1, S5200-1, S52, S6200-1, S9220-1, S7240-1, S7210-1, S6230-1, S6240-1, S3250-1, S0290-1, S5210-1, S4230-1, S1220, S9230-1, S0200-1, S3270, S2210-1, S8260-1, S4240-1, S5250-1, S6220-1, S621, S6210-1, S4220-1, S5260-1, S0270-1, S7230-1, S8230-1, S8220-1, S422-3, S5241, S327, T0210, T0220-1, T0240, T0230-1, T0281, T0250-1, T0260, T0280, T029, T0290, 1, T10-0, 1, T08-0, 1, T841, T825, T864, T902, T911-2, 8, T92, 3, T921, T931, 2, T941, T1420-1
Amputation	(receiptcode) 150051610, 150051710, 150051810
Atrial fibrillation	(icd10code) I480-2, I489
Valvular heart disease	(icd10code) A520, I340-2, 8, I060-2, 9, I38, I050 -2, 8-9, I080-3, 8-9, I091, I070-2, 8, 9, I350-2, I358-9, I360-2, 9, I489, Q230-3, 8, Q222, 4, 8, Q252-3, Q244, Z952, 4, Z988
Liver disease	(icd10code) A188, A064, A527, B251, B008, B150, 9, B162, 9, B171-2, 8-9, B181-2, 9, B190, 9, B268, B279, B338, B581, B659, B660-1, 3, B670, 5, 8, B838, B89, C183, C220-4, C227, 9, C240, 8, C772, C861, C787, D134-5, D175, D180, D376, D684, D868, D612, D848, E106, E116E130-4, E135-7, E139 E146, E550, E722, E740, 3, 6, E802, E831, E888, H02, I820, I81, I728, 748, J632, K650, K701-4, 6, 9, 10, K711-3, 7-9, K720, 1, 9, K730-2, 8-9, K740-1, 3-6, 8, K805, K750, 2-4, 8-9, K762-5, 7-9, K830-1, 8, K91, L811, O266, O984, O997, P150, P788, P353, P592, P788, P002, Q266, Q441-, 2, 4-7, R160, R609, R945, R18, S351-2, S361, 7, S3610-1, Z904, Z205, Z526, Z861, Z488, Z944
Diuretics	(receiptcode) 620003807, 620004182, 620004650, 620008863, 620008864, 620267805, 622098801, 622327400, 622750700, 622750800, 642130005, 642130006, 610461153, 610461156, 612130039, 612130267, 612130342, 620003083, 620003560, 620003591, 620004443, 620004558, 620004615, 620004914, 620004915, 620006149, 620008345, 620266102, 620266110, 620266112, 620266119, 620266132, 620266138, 620266148, 620266304, 620266305, 620266409, 620266412, 620266413, 622315800, 612130169, 620006051, 610433078, 610453012, 610454048, 612130353, 620270703, 622161501, 622161502, 622721300, 622721400, 610432034, 610432035, 622601001, 622601101, 622609402, 622609502, 612140164, 612130313, 612130314, 642130019, 610431025, 610433104, 612130071, 612130220, 620000167, 620000168, 620000339, 620000340, 620003182, 620003238, 620003440, 620003612, 620003836, 620004041, 620004042, 620004043, 620004758, 620004759, 620005097, 620005221, 620005550, 620006110, 620007070, 620009199, 620269701, 620269703, 620269809, 620269833, 620270101, 620271902, 620272101, 620272102, 621782401, 622077601, 622109301, 622531501, 622531601, 622531701, 622548901, 622567401, 620007146, 620007548, 620004938, 620008677, 620268501, 610463125, 612140560, 612140561, 612140674, 612130207, 620004907, 620005049, 620005050, 620005953, 620006050, 620006106, 620007003, 620009400, 620009401, 620009430, 620262802, 620263001, 620263201, 620263401, 620263901, 620264101, 622142401, 622142402, 622383401, 612130126, 620008670, 620264701, 622142501, 622142502, 622280101, 620008715, 622007201, 622174301, 622281901, 622537701, 622694301, 622694401, 622694501, 612130050, 620002860, 620002861, 620007900, 621859002, 621859102, 622065501, 622065601, 622065701, 622065801, 622184801, 622721100, 620004919
SGLT2 inhibitors	(receiptcode) 622306601, 622306701, 622401201, 622401301, 622360601, 622341901, 622342001, 622336801, 622340101, 622335701, 622335801, 622655001, 622655101, 622625702, 622573601
Statins	(receiptcode) 610443013, 610443014, 622076401, 622076501, 622098401, 622098501, 622107601, 622107701, 622110401, 622110501, 622126901, 622127001, 622143801, 622143901, 622152001, 622152101, 622161801, 622161901, 622165601, 622165701, 622167601, 622167701, 622170101, 622170201, 622180601, 622180602, 622180701, 622180702, 622186601, 622186701, 622187601, 622187701, 622204801, 622204901, 622217101, 622217201, 622241301, 622241401, 622244801, 622244901, 622252001, 622252101, 622280701, 622280801, 622302401, 622302501, 622360101, 622475000, 622475100, 610462015, 610462016, 612180263, 620000052, 620000053, 620000054, 620000169, 620000174, 620000175, 620000176, 620000177, 620000178, 620002163, 620002166, 620002736, 620002880, 620009322, 620009323, 620009324, 621524102, 621524402, 621533501, 621533801, 621533901, 621534003, 621534101, 621534204, 621534301, 621623603, 621635202, 621643301, 621643401, 621643501, 621643601, 621675101, 621948701, 621955001, 621955003, 622052801, 622075801, 622099101, 622116802, 622128201, 622169901, 622315400, 622315500, 620000422, 620000423, 622136401, 622239201, 622239301, 622268001, 622268101, 622268201, 622269101, 622269201, 622270001, 622270101, 622271801, 622271901, 622273101, 622273201, 622273301, 622274901, 622275001, 622275101, 622276301, 622276401, 622276501, 622280201, 622280301, 622280401, 622280501, 622280601, 622282201, 622282301, 622283701, 622283801, 622285001, 622285101, 622286201, 622286301, 622286401, 622287601, 622289501, 622289601, 622291801, 622291901, 622292001, 622292301, 622292401, 622292501, 622293301, 622293401, 622294301, 622294401, 622294501, 622296001, 622296101, 622296201, 622297101, 622297201, 622298001, 622298101, 622298201, 622299001, 622299101, 622302801, 622302901, 622304601, 622304701, 622304801, 622304901, 622359101, 622365801, 622372401, 622387001, 622387601, 622406901, 622419701, 622419801, 622419901, 622421601, 622421701, 622421801, 622431401, 622434301, 622434401, 622457701, 622457801, 622457901, 622464901, 622465101, 622512001, 622512101, 622512201,622522101, 622522201, 622522301, 622524501, 622524601, 622524701622528901, 622529001, 622568601, 622604001, 622604101, 622604201,622615600, 622615700, 622615800, 622615900, 622665901, 622666001, 622666101, 622691800, 610470012, 610470013, 610470014, 621934901, 621935001, 621964101, 621964201, 621964301, 621964401, 621964501, 621964601, 622015101, 622015201, 622015301, 610454084, 610454085, 612180264, 612180265, 620000013, 620000014, 620000070, 620000103, 620000104, 620000105, 620000106, 620000107, 620000108, 620000109, 62000011-4, 620000141, 62000015-3, 62000015-9, 620000160, 620000163-4, 620000171, 620002108-9, 620002798-9, 620002800,620002802, 620004038, 620006681, 620008053-6, 621521301, 621521401,621523101, 621523201, 621525701, 621525801, 621528501, 621528602,621528702, 621528801, 621528901, 621529001, 621529101, 621531001,621531101, 621531703, 621532401, 621532501, 621532601, 621532902,621533002, 621533101, 621533201, 621533601, 621639001, 621639101,621639701, 621639801, 621694001, 621752501, 621981401, 621981403,622062701, 622139600, 622321900, 622606601, 622606701, 620002477,620002478, 622441101, 622441201, 622537301, 622537401, 622571801,622571901, 622572801, 622572901, 622575201, 622575301, 622575401,622575501, 622575601, 622575701, 622577901, 622578001, 622578101,622578201, 622578401, 622578501, 622578601, 622578701, 622578801,622581601, 622581701, 622581801, 622581901, 622582001, 622582101,622582501, 622582601, 622582701, 622582801, 622584701, 622584801,622586001, 622586101, 622586201, 622586301, 622588801, 622588901,622589001, 622589101, 622590101, 622590201, 622590301, 622590401,622591701, 622591801, 622591901, 622592001, 622592501, 622592601,622592701, 622592801, 622592901, 622593001, 622593101, 622593201,622595301, 622595401, 622598301, 622598401, 622598501, 622598601,622599201, 622599301, 622599401, 622599501, 622600301, 622600601,622600701, 622600801, 622600901, 622601201, 622601301, 622601401,622601501, 622605001, 622605101, 622605201, 622605301, 622605401,622640801, 622640901, 622644801, 622644901, 622660001, 622660101,622692400, 622692500, 622692600, 622692700, 622803401, 622803501,622803701
Antiplatelets	(receiptcode) 620003468, 620003469, 622401801, 622401901, 622405801, 622405901 622406101, 622406201, 622407701, 622407801, 622411301, 622413301, 622413401, 622413501, 622413601, 622413701, 622414401, 622414501, 622416201, 622416301, 622418201, 622418301, 622420101, 622420201, 622420301, 622420501, 622420502, 622420601, 622420602, 622422201, 622422301, 622424101, 622424201, 622424301, 622425001, 622425101, 622425501, 622425601, 622425701, 622427501, 622427601, 622428501, 622429501, 622429601, 622429701, 622430501, 622430601, 622430701, 622431201, 622431301, 622431501, 622431601, 622432801, 622432901, 622433001, 622433101, 622433201, 622433301, 622433401, 622434501, 622434601, 622435501, 622435601, 622438201, 622438301, 622439501, 622439601, 622439801, 622439901, 622475400, 622475500, 622641801, 622472201, 622472301, 610407355, 610461073, 613390001, 613390007, 613390015, 620003275, 620004293, 620004843, 620005039, 620005788, 620005812, 620008315, 620008318, 620008330, 620009246, 620814301, 620814502, 620814506, 620814511, 620814512, 620814521, 620814523, 620814528, 620814531, 620814533, 620814534, 620814543, 620814601, 621374002, 622336601, 622336701, 622452301, 622486701, 622658901, 610444003-4, 610444026, 610444051, 610444056-9, 610444127-9, 610444130-3, 61044414-1, 610453021, 620000049, 620000050, 620002105, 620003450-1, 620003610-1, 620005094, 62000932-1, 621312902, 621313101, 621313301, 621313502, 621313601, 621313702, 621313801, 621314001, 621314101, 621314301, 621314601, 621314802, 621314901, 621315002, 621315201, 621315401, 621418301, 621418401, 621418501, 621418601, 621418701, 621418802, 621638701, 621721201, 621721301, 621971201, 62197130, 622088701, 622088801, 622119801, 622226901, 622227001, 622256201, 622256301, 622315200, 622315300, 622334101, 622334201, 622344001, 622344101, 622346301, 622347901, 622361301, 622361401, 622373001, 622373101, 622384101, 622384201, 622501801, 622501901, 610406235, 610406281, 610406400, 620003616, 620008722, 620314401, 621251701, 622161301, 622161302, 622314300, 610463123, 610463181, 610463184-5, 610463190, 610463192, 610463195, 613390023-4, 620002116, 620002119, 621481301, 621481401, 621481501, 621481601, 621481801, 621481903-4, 621482001, 621482101, 621482201, 621482302, 621526401, 621530401, 621639901, 621640001, 622147201, 610453061, 610463047, 620000024, 620002509, 620002538, 620002881, 620004095, 621418905, 621481101, 621522101, 621623701, 621694102, 622738800, 622290301, 622795301, 622341601, 610432021, 613390026-7, 621927101, 621927201, 621930601, 621930701, 621935102, 621935202, 621936701, 621937001, 621937801, 621939201, 621939301, 621940701, 621940801, 621941901, 621942001, 621942801, 621946101, 621946201, 621948201, 621948301, 621949501, 621949601, 621951401, 621951501, 621953101, 621953201, 621954802, 621954902, 621960801, 621960901, 621961001, 621961301, 621961401, 621961501, 621961601, 621961701, 621961801, 621961901, 621962001, 621962101, 621974101, 621992001, 622089801, 622089901
DOACs	(receiptcode) 622043301, 622043401, 622224901, 622225001, 622080901, 622081001, 622375201, 622576001, 622576101, 622576201, 622068301, 622068401, 622449101, 622449201
Calcium channel blockers	(receiptcode) 610470001-2, 622230401, 622230501, 622233401, 622233501, 622236201, 622236301, 622240001, 622240002, 622240101, 622240102, 622241101, 622241201, 622243401, 622243501, 622247001, 622247101, 622251401, 622251501, 622253701, 622253801, 622257801, 622257901, 622258201, 622258301, 612170707, 612170708, 612170709, 612170710, 620003886, 620003887, 620005904, 620005905, 620007817, 620007818, 620007819, 620007820, 620007822-3, 620007826-9, 62000783-1, 620007833-4, 620007836-9, 620007840-9, 620007850-4, 620007856-7, 620007860-8, 620007870-9, 620007880-6, 620008040-1, 621847203, 621847303, 621852302, 621852402, 621853103, 621853203, 621855203, 621857103, 621931301, 621931401, 621931901, 621932001, 621934401, 621934402, 621934403, 621934501, 621934502, 621934503, 621936803, 621936901, 621936903, 621937301, 621937401, 621938501, 621938601, 621941201, 621941301, 621941601 621941701, 621944301, 621948401, 621948501, 621949101,621949201, 621951201, 621951301, 621952603, 621952605, 621952703, 621952705, 621954601, 621954603, 621954701, 621954703, 621956801, 621956901, 621957301, 621957401, 621958901, 621959001, 621959101, 621959201, 621959301, 621959401, 621959501, 621959601, 621959701, 621959801, 621973001, 621973101, 621982905, 621985501, 621985601, 621991101, 621991201, 622002701, 622002801, 622018401, 622018501, 622029501, 622029502, 622029601, 622029602, 622137601, 622141201, 622143001, 622143101, 622163901, 622180801, 622183801, 622184701, 622187801, 622187901, 622189801, 622189901, 622193201, 622198101, 622201301, 622201401, 622202301, 622202901, 622207401, 622207501, 622208801, 622211901, 622213802, 622215001, 622216201, 622216301, 622219501, 622219601, 622221101, 622221501, 622233601, 622234501, 622239501, 622243801, 622248901, 622249601, 622249701, 622249801, 622253901, 622257701, 622260701, 622260901, 622261701, 622269701, 622282601, 622284001, 622284002, 622284101, 622285601, 622287301, 622288501, 622290901, 622298501, 622301601, 622309600, 622309700, 622309800, 622360701, 622360801, 622360901, 622439701, 622689200, 620000040, 620000041, 620005002, 620005102, 610421346, 612140718, 612140719, 610407147, 610407148, 610407151, 610407152, 620009318, 620009319, 621934601, 621934701, 622022201, 622022301, 622024201, 622024301, 622039202, 622039302, 622284801, 622486601, 622491201, 622496301, 622506001, 622519901, 610412073, 610412184, 610453090, 612170598, 620002053, 620002543, 620003619, 620003620, 620004477, 620006155, 620006932, 620008718, 620307001, 620307006, 620307010, 620307012, 620307026, 620307403, 620307601, 620307701, 62030770, 621575701, 621575702, 621579602, 621936201, 621941402, 621945702, 621960602, 621992201, 622010101, 622035902, 622082701, 622106802, 622314900, 622315100, 622726000, 622726200, 610454080, 610454081, 612140483, 612190066, 612190103, 612190234, 612190236, 612190247, 612190250, 620003617, 620003618, 620004539, 620005003, 620005004, 620005005, 620005006, 620006747, 620294901, 620295101, 620295601, 620295802, 620347801, 620348001, 620348401, 620349001, 620349101, 620349302, 620349501, 620349901, 620350201, 621256201, 621960702, 621963901, 621963902, 622319700, 622319800, 610463138, 610463211, 612170631, 612170670, 620007010, 621470601, 621470703, 621470902, 621471001, 621471103, 622320100, 610412129, 610412142, 610412143, 610422193, 610422194, 610422214, 610422215, 610422225, 610422226, 610461204, 610461205, 612170632, 612170633, 620326701, 620326801, 620327001, 620327202, 620327602, 620328101, 620328201, 620328401, 620328602, 620329002, 621253401, 610406218, 610406219, 610406284, 610421320, 610421321, 610421322, 610433041, 612170539, 612170540, 612170547, 612170610, 612170647, 612170668, 612170676, 612170677, 612170683, 612170692, 612170693, 612170698, 612170699, 612170703, 612170705, 612170706, 620001881, 620002078, 620002079, 620002080, 620002086, 620002087, 620002514, 620002723, 620002724, 620002758, 620002759, 620002762, 620003132, 620003133, 620003442, 620003529, 620003952, 620004014, 620004015, 620004016, 620004437, 620006003, 620006004, 620006128, 620006819, 620007014, 620007015, 620007016, 620008034, 620008035, 620008036, 620008037, 620317401, 620317603, 620318401, 620318701, 620318801, 620318901, 620319101, 620319704, 620319901, 620320201, 620320301, 620320401, 620320601, 620322401, 620323403, 621252701, 621621501, 621621601, 621637101, 621675601, 621675701, 621685101, 621685201, 621685401, 621733201, 621838103, 621838203, 621838303, 622029402, 622077702, 622320500, 622320600, 622320700, 622636801, 622636901, 622637001, 622727100, 622727200, 622727300, 622727500, 622727600, 622727700, 610463141, 610463142, 612140495, 612140496, 620000080, 620000081, 620000082, 620002757, 620005523, 620005524, 621469201, 621469301, 621469401, 621526601, 621526703, 621526803, 621685001, 622072601, 612140697, 612140698, 612140699, 610407003, 610407004, 610407010, 610407011, 620002700, 620002701, 621671702, 621671802, 612170662, 612170663, 612170664, 620003900, 620003902, 620003903, 620003905, 620003951, 620004047, 620004048, 620004049, 620004050, 620004051, 620004052, 620004053, 620004054, 620004055, 620004056, 620004057, 620004058, 620004059, 620004060, 620004061, 620004062, 620004063, 620004064, 620004066, 620005429, 620005551, 620005552, 620005553, 620005554, 620005555, 620005556, 620006664, 620006665, 620007997, 620008063, 620008064, 620008065, 620008066, 620008067, 620008068, 620009434, 621726901, 621727001, 621730802, 621730902, 621736902, 621739203, 621739403, 621739601, 621739702, 621751201, 621751301, 621783103, 621793502, 621793602, 621867101, 621948101, 621948102, 621953901, 621985102, 621985202, 622050101, 622050201, 622050301, 622058602, 622126201, 622126301, 622323500, 622728200, 622728300, 620004581, 620004582, 620004629, 620007085, 620309001, 620309101, 620309102, 622015601, 612140554, 612140555, 612140556, 620002012, 620002013, 620002044, 620002045, 620002132, 620002133, 620002134, 620002136, 620002138, 621623001, 621623101, 621630801, 621630901, 621632401, 621632501, 621634701, 621634801, 621638003, 621638004, 621640901, 621641001, 621641101, 621641201, 621941502, 621960402
Insulin	(receiptcode) 620000265, 620008897, 620008907,620008908, 620008909, 620008932, 622114401, 640407220, 640422068, 640422074, 640453021, 642490059, 642490107, 620008893, 620008894, 620008895, 621926901, 622252701, 629905601, 629905701, 629905801, 640451038, 640451040, 640451041, 621911101, 621911201, 621911301, 620007460, 620008916, 622642701, 629906701, 629906801, 629906901, 629907001, 640451027, 640451028, 640451029, 629907301, 629907401, 629907501, 620000266, 620008898, 620008910, 620008911, 620008912, 620008933, 622114501, 640407221, 640412085, 640422069, 640453022, 642490061, 642490123, 620002441, 620002444, 620007459, 622450901, 622451001, 620000204, 620000205, 620000267, 620000269, 620000270, 620000271, 620008899, 620008913, 620008914, 620008915, 620008934, 620008936, 622114601, 640406239, 640407222, 640412082, 640412084, 640422067, 640453023, 642490121, 620000447, 620000448, 620008896, 621973201, 621973301, 620002439, 620002440, 620002442, 620002443, 620007461, 620007462, 620000442, 620000443, 620002445, 620007536, 620008942, 620008943, 620008945, 622440701, 622410901, 622411001, 622484801, 622198901, 622199001, 620005900, 620005901, 620008952 ,620008953, 621927001 ,629900701 629907601
Oral antidiabetic agents (other than SGLT2 inhibitors)	(receiptcode) 613960017, 620000048, 620009209, 620002029, 620002030, 620002031, 620002032, 620003568, 620003947, 620003948, 620004482, 620006891, 620873202, 620873301, 620873402, 620873702, 622005802, 622141302, 622143402, 622169102, 622313200, 622740400, 622740500, 620006030, 610412056, 610433079, 610441043, 613960002, 613960003, 613960038, 613960039, 613960079, 620003159, 620003160, 620003604, 620006890, 620871407, 620871601, 620871907, 620872001, 620872002, 620872003, 620872004, 620872009, 620872016, 622009802, 622013401, 622018801, 622018802, 622036001, 622036002, 622075601, 622103201, 610443002, 610443003, 621982701, 621997001, 621997101, 621998701, 621998801, 621998901, 621999001, 621999301, 621999401, 621999701, 621999801, 622000601, 622000701, 622001701, 622001801, 622004701, 622004702, 622004801, 622004802, 622005501, 622008701, 622008801, 622009901, 622010001, 622011401, 622011501, 622011601, 622011701, 622013501, 622013601, 622016001, 622016101, 622017301, 622017401, 622017501, 622017901, 622018001, 622020903, 622021003, 622021801, 622021901, 622022001, 622022101, 622023501, 622023601, 622025201, 622025301, 622025801, 622025901 622026501, 622026601, 622029901, 622031401, 622031501, 622033001, 622033101, 622033201, 622033701, 622033801, 622035701, 622035801, 622037901, 622038001, 622058801, 622059001, 622059002, 622059102, 622088301, 622088401, 622114701, 622114801, 622118501, 622122201, 622122301, 622127301, 622127401, 622127501, 622128101, 622137701, 622141101, 622144001, 622159301, 622169301, 622176301, 622177501, 622186201, 622187301, 622190001, 622190002, 622193301, 622194901, 622202201, 622202801, 622205101, 622205501, 622208901, 622211501, 622217701, 622219701, 622221001, 622222001, 622242001, 622246801, 622252501, 622254701, 622271101, 622271201, 622271301, 622313300, 622338501, 622338601, 622338701, 622631401, 622636501, 622636601, 613960060, 620003277, 620003452, 620004502, 620005979, 610444147, 610463145, 613960032, 620004480, 620005570, 620009133, 621676001, 621974701, 622070801, 622242501, 622412701, 622417101, 622417201, 622421101, 622421201, 622421901, 622422001, 622424401, 622424501, 622427201, 622427301, 622432601, 622432701, 622436301, 622438401, 622438501, 622448601, 622466601, 610432040, 610432041, 621990901, 621991001, 622041202, 622041302, 622041402, 622041502, 622042901, 622043001, 622045201, 622045301, 622045401, 622046801, 622046901, 622047701, 622047801, 622049901, 622050001, 622053101, 622053201, 622053801, 622055801, 622055901, 622056001, 622056101, 622059201, 622059301, 622061001, 622061401, 622061501, 622061601, 622061701, 622062302, 622062401, 622062402, 622063001, 622063101, 622063201, 622063301, 622065101, 622065201, 622065301, 622065401, 622066201, 622066301, 622071701, 622071801, 622071901, 622072001, 622078301, 622078401, 622079101, 622079201, 622081801, 622081901, 622144601, 622144701, 622147301, 622147401, 622147501, 622147601, 622155701, 622155801, 622156001, 622156901, 622157001, 622159401, 622159501, 622163301, 622163401, 622164401, 622166801, 622166901, 622167201, 622167301, 622172101, 622172201, 622175401, 622175501, 622175601, 622175701, 622182401, 622320800, 622320900, 622741400, 622795901, 622796001, 622048401, 622048501, 621986301, 621986401, 613960081, 613960082, 620005359, 620005360, 620009286, 620009287, 620009288, 620009289, 620009290, 620009291, 620009292, 620009293, 620009294, 620009295, 620009296, 620009297, 621784902, 621785002, 621896402, 621896502, 621937101, 621937201, 621942102, 621942201, 621942202, 621958701, 621958801, 622008501, 622008502, 622008601, 622008602, 622302301, 610406390, 610406391, 620002120, 620002121, 620002812, 620002813, 620002825, 620002826, 620002827, 620002835, 620002836, 620002840, 620002841, 620002842, 620002843, 620002845, 620002846, 620002847, 620004045, 620004046, 620004069, 620004070, 620004071, 620004072, 620004073, 620004074, 620005557, 620005558, 620005559, 620005560, 620005561, 620005562, 620005563, 620005564, 620005565, 620005566, 620006682, 620006683, 620008071, 620008072, 620008073, 620008074, 620008075, 620008076, 620008726, 620008727, 620008728, 620008729, 621665301, 621665401, 621673501, 621673601, 621683401, 621683501, 621689001, 621689101, 621689303, 621689403, 621690203, 621690303, 621690402, 621690502, 621690901, 621691001, 621691101, 621691201, 621691501, 621691601, 621943301, 621943401, 621953301, 621953401, 622090001, 622090101, 622662501, 622662601, 622662701, 622662801, 622741100, 622741200, 620003127, 620003128, 620003129, 622426601, 622426701, 622432501, 622544301, 622544401, 622544501, 622560201, 622560301, 622560401, 622628601, 622628701, 622628801, 622664101, 622664201, 622664301, 610432026, 610432027, 610432032, 610432033, 622119301, 622119401, 622196601, 622196701, 622230001, 622230101, 620001907, 620001908, 622462401, 622462501, 622515201, 622515301, 622518101, 622518201, 622520901, 622521001, 622523301, 622523401, 622525301, 622525401, 622040901, 622041001, 622813401, 622813501, 622053601, 622671001, 622201701, 621986001, 621986101, 621986201, 622448901, 622449001, 622245601, 622245701, 621950901, 621951001, 621951101, 621970601, 621970701, 621970801, 622277501, 622288401, 622182601, 622660601, 622415401, 622415501, 622699501, 621980701, 622093501, 622654401, 622654501, 622517101, 622450301, 622450401, 622086001, 622086101, 622655001, 622655101, 622625702, 622573601, 622038301, 622038401, 622229001, 622406001, 629907701, 629907801, 629907901, 622442201, 622267001, 621974801
Beta blockers	(receiptcode ) 612120004, 612120148, 610412007, 610461002, 610461003, 612120318, 612120319, 612120320, 612120321, 612120324, 612120330, 612120335, 612120336, 612120347, 612120348, 612120356, 620002066, 620002067, 620004844, 620006586, 620006602, 620008281, 620008283, 620009609, 620009610, 620254403, 620254406, 620254407, 620254410, 620254411, 620254412, 620254417, 620254418, 620254419, 620254424, 620254426, 620254501, 620254509, 620254517, 620254522, 620254527, 620254532, 620254544, 620254553, 620254554, 620254560, 621974401, 621974402, 612140481, 620250701, 610406002, 610406009, 610406028, 610406157, 610406158, 610431115, 612120224, 612120225, 620003512, 620255102, 620255302, 620255401, 620255403, 620255408, 620255417, 620255420, 622052102, 622055502, 622062802, 622071301, 622071401, 622108802, 622310500, 622310600, 622719000, 622719100, 610454035, 612120119, 620003178, 620004527, 620004593, 620252204, 620252207, 620252210, 621770001, 610462039, 610462040, 610463009, 610463010, 610463013, 612140702, 612140703, 620000005, 620000006, 620002018, 620002708, 620002709, 621470101, 621470201, 621470301, 621470401, 621520501, 621520601, 621681901, 621682001, 622049001, 622049101, 622095501, 622095601, 622206801, 622206901, 622312800, 622355501, 622064802, 622355601, 622380901, 622381001, 622479201, 622479301, 622481401, 622481501, 622490801, 622490901, 622494301, 622494401, 622496101, 622496201, 622500301, 622500401, 622504201, 622504301, 622644401, 622644501, 622644601, 622644701, 622725200, 622725300, 622725400, 610444066, 610444067, 610444073, 610444080, 612140695, 612140696, 620003441, 621248601, 621248701, 621248801, 621248803, 621248901, 621249001, 621249102, 621249103, 621402601, 621402701, 621720801, 621977100, 621977101, 622725000, 610421339, 610421340, 612120226, 612120227, 610453089, 612140521, 622264101, 622264201, 622669401, 610453017, 610453018, 610453097, 610453122, 610453123, 610453124, 610453125, 610453127, 612120266, 612120267, 620001874, 620001875, 620256101, 620256201, 621399302, 621399401, 621399501, 621399602, 621399902, 621400001, 621400301, 621400302, 621400401, 621400502, 621400601, 621400901, 621573401, 621573402, 622060401, 622060402, 622068001, 622068002, 622267901, 622271001, 622284601, 622289401, 622289402, 622296701, 622296801, 622339301, 622591001, 622591101, 622591201, 622719200, 622719300, 612120029, 612120031, 612140450, 612140451, 620004559, 620005077, 620008697, 620253703, 620008582, 612120016, 612120017, 612120124, 620004453, 620004586, 620005031, 620005972, 620252707, 620252712, 620252713, 620293401, 622322800, 610463027, 610463100, 610463187, 610463188, 612140700, 612140701, 620002034, 620002035, 621469501, 621469601, 621469801, 621469901, 621470001, 621633801, 622097101, 622323000, 622323100, 610407019, 610407020, 610407021, 610406167, 610406168, 612140128, 612140129, 612140327, 612140328, 612140552, 612140670, 612140671, 612140684, 612140685, 620003565, 620008781, 620290102, 620290104, 620290106, 620290111, 621366703, 621366706, 621366718, 621366720, 621965402, 622105701, 622117102 ,622324500, 622723500, 612140158, 612140159, 612140661, 612140662 ,612140693, 620289301, 620289701, 612140468
Erythropoiesis-stimulating agents	(receiptcode) 622068801, 622068901, 622069001, 622069101, 622069201, 622069301, 622069401, 622607501, 640407002, 640407003, 640407004, 640407005, 640408136, 640462046, 640462047, 640462048, 640462049, 640462050, 640462051, 640462052, 643990105, 643990106, 621972301, 621972401, 621972501, 621972601, 621972701, 621972801, 621433101, 621433201, 621997901, 640463031, 640463032, 640463033, 640463034, 640463037, 640463038, 640463039, 640463040, 640463041, 640463042, 620004877, 620004878, 620004879, 620004880, 620004881, 620004882, 620004883, 620009109, 620009110, 620009111, 620009112, 620009113, 620009114, 620009115, 621987701, 621987801, 621987901, 621988001, 621988101, 621988201 ,621988301, 621988401, 622199501, 622199601, 622199701, 622199801, 622199901, 622200001, 622200101, 622200201, 622293801, 622681601, 622681701, 622681801, 622681901, 622682001, 622682101, 622682201, 622682301, 622682401, 629902301, 629902401, 629902501, 629902601, 629902701, 629902801, 629902901, 629903001, 629903101, 629904001, 629904101, 629904201, 629904301, 629904401, 629904501, 629904601, 629904701, 629904801, 629900801, 629900901, 629901001, 629901101, 629901201, 629901301, 629901401, 629901501, 629901601
Charcoal absorbents	(receiptcode) 610444045, 613920083, 620002027, 620002028, 620002153, 620002154, 620009308, 620009309, 620853401, 621320601, 621633002, 621633102, 622585201, 622739400, 622739500
Sodium bicarbonate	(receiptcode) 620476624, 610409223, 610409225, 610409227, 610431057, 612340028, 612340106, 612340107, 612340108, 612340109, 612340110, 612340112, 612340117, 612340143, 620000660, 620000662, 620000664, 620000666, 620002046, 620006672, 620476415, 620476427, 620476463
Potassium absorbents	(receiptcode) 622796601, 622796701, 610408579, 610444005, 610463066, 612190191, 612190342, 620003443, 620003538, 620006880, 620006881, 620008730, 620009253, 620356905, 620356906, 620356908, 621261101, 622140001, 622189501, 610407233, 620004980, 620355001, 622003501, 622729200
Phosphorus absorbents	(receiptcode) 622442301, 622442401, 622651201, 622651301, 610470003, 610470004, 622070102, 622070202, 622149201, 622516401, 610432025, 610462045, 610462046, 620002705, 620009351, 620009352, 621668203, 621977701, 621977801, 622102601, 622102602, 620008560, 620008561, 622068101, 622068201, 622552001, 622552101, 622620601, 622620701, 622623301, 622623401, 622626101, 622626201, 622627101, 622627201, 622635901, 622636001, 622638601, 622638701, 622642901, 622643001, 622664601, 622664701,622667501, 622667601, 622700601, 622700701, 622702201, 622702301
Vitamin D	(receiptcode) 613110001, 613110021, 613110024, 613110025, 613110029, 613110031, 613110032, 613110034, 613110036, 613110075, 613110078, 620000699, 620004951 ,620006882, 620006884, 620675301, 620675401, 620675904, 620675906, 620675910, 620675913, 620675922, 620675928, 620675929, 620675931, 620676201, 622074901, 622083201, 622310300, 622310400, 622737600, 622737700, 622042502, 610406073, 610406074, 610406083, 610406084, 610406085, 610406089, 610406090, 610406094, 610406207, 610406252, 610406341, 610461109, 613110017, 620003088, 620678104, 620678110, 620678130, 620678136, 621576003, 622737800
Iron	(receiptcode) 610406259, 610406262, 610407094, 610407098, 613220038, 613220039, 620004564, 620006090, 620007053, 620007054, 620730201, 620730403, 621307601, 621307701, 622288601, 622738100, 622738200, 613220022, 620729301, 620005924, 613220015, 620006952, 613220018, 613220023, 620006032, 620729101
Beta2 agonists	(receiptcode) 620006996, 620409401, 620409501, 620409701, 612220290, 620008713, 620008714, 620402601, 620004220, 620004221, 620004223, 620004224, 620004226, 620004227, 620004228, 620004229, 620004230, 620004231, 620004232, 620004233, 620004234, 620004235, 620004236, 620004237, 620004240, 620004242, 620004243, 620005746, 620005749, 620005750, 620005751, 620005752, 620005753, 620005754, 620008256, 621720401, 621720501, 621761601, 621761701, 621761801, 621762601, 621762701, 621762801, 621763001, 621763101, 621951801, 621951901, 621952001, 622049201, 622049301, 622049401, 622148501, 622148601, 622148701, 660421115, 660421116, 660421117, 612220293, 620002544, 620002545, 620003986, 620005017, 620005018, 620006979, 620006980, 620008720, 620008721, 620404101, 620404601, 621745601, 620007065, 620007066, 620007478, 620399501, 620399701, 620004932, 620004933, 621269105, 610406290, 612250010, 620008723, 620008724, 610407409, 610407410, 610407438, 610407455, 610461088, 620002152, 620003631, 620003632, 620003633, 620003634, 620005933, 620006147, 620006566, 620008777, 621369604, 621369613, 621369616, 621369711, 621369714, 621369723, 621369806, 621369813, 621957602, 621965302, 620004905, 612220511, 612220044, 620008586, 662220014, 662220006, 662220007, 620006846, 620410001, 622096401, 620001944, 660462003, 660462004, 622180901, 622277401, 620008998, 620009062, 620403601, 660412002, 660422029, 662220027, 660433071, 620000403, 620000404, 620002626, 620004835, 621903102, 621903201, 621903202, 622308301, 660463035, 660463036
Corticosteroid inhalers	(receiptcode) 620004888, 620004889, 620004890, 622057501, 622552201, 622552301, 620000453, 621512601, 621572201, 660421112, 660421113, 660421114, 660451012, 660451013, 660451016, 660462011, 620004366, 620004367, 620005290, 620005291, 620005292, 622701801, 622701901, 660462001, 660462002, 620004885, 620004886, 620004887, 620007565, 620007566, 620007567, 620009104, 621781401, 621781501, 621781601, 621829501, 621829601, 621829701, 621895501, 621981201, 621981301, 622279201, 622279301, 622375501, 622375601, 622277901, 622278001, 622278101, 622278201, 621950701, 621950801, 622700201, 622700301, 622702701, 622816601
Dialysis	(receiptcode) 140007710, 140007910, 140008170, 140029850, 140033770, 140036710, 140051010, 140051110, 140052570, 140052810, 140052970, 140053670, 140057810, 140057910, 140058010, 140058410, 140058510, 140058610, 140058770, 140058870, 140058970, 140059070, 140059170, 140059310, 620004183, 620004184, 620004185, 620004186, 620004187, 620004188, 620005155, 620005156, 620005157, 620006312, 620007210, 620007213, 620007218, 620007308, 620007430, 620007544, 620008806, 620008808, 620008825, 620009138, 620009139, 620009140, 620009151, 620009154, 620009157, 620009166, 620009173, 620009175, 620009176, 620009177, 620009178, 620009180, 620009182, 620009184, 620009185, 620009186, 620009187, 620009189, 620009195, 620009543, 620009544, 620822001, 620822301, 620822901, 620823001, 620823201, 620830401, 621317301, 621317501, 621317601, 621317701, 621318101, 621318401, 621318501, 621515201, 621561701, 621561801, 621653101, 621653201, 621728101, 621728201, 621989301, 622054501, 622054701, 622083901, 622131301, 622131401, 622181701, 622300501, 622301001, 622396501, 622396701, 622456001, 622466701, 622466801, 622466901, 622471101, 622533201, 622533301, 710010654, 710010828, 710010829, 720110000, 732670000, 732680000, 732690000, 734000000, 734010000, 734020000, 734030000, 737890000, 738100000, 738110000
Malignant cancer	B210-2, C000-4, C006, C008-9, C01, C020-2, C029, C030-1, C039, C040-1, C049, C050-2, C059, C060-2, C069, C07, C080-1, C089, C090-1, C099, C100-4, C109, C113, C119, C12, C130-2, C139, C140, C150-5, C158-9, C160-5, C169, C170-2, C179, C180-7, C189, C19, C20, C210-1, C220-4, C227, C229, C23, C240-1, C248-9, C250-4, C258-9, C261, C269, C300-1, C310-3, C319, C320-3, C329, C33, C340-3, C349, C37, C380-4, C400-3, C410-4, C419, C430-9, C440-7, C449, C450-2, C459, C469, C470-6, C479, C480-2, C490-6, C499, C500-6, C509, C510-2, C519, C52, C530-1, C538, C539, C540-3, C549, C55-6, C570, C579, C58, C600-2, C609, C61, C620-1, C629, C630-2, C637, C639, C64-6, C670-7, C679, C680-1, C690-6, C700-1, C710 -9, C720-1, C723-5, C729, C73, C740-1, C749, C750-5, C760-5, C770-5, C778-9, C780, C782 -8, C790-5, C798-9, C800, C809, C817, C819, C820-1, C823-4, C826-7, C829, C830-1, C833, C835, C837-8, C840-1, C844-8, C851-2, C859, C860, C862-3, C865-6, C880, C882-4, C900-3, C910-1, C913-9, C920-5, C927-9, C930-1, C933, C939, C940, C942-4, C946-7, C950-1, C959, C960, C962, C964-6, C968, D000-2, D010-4, D019, D020-3, D030-1, D033, D036-9, D040-7, D049, D050-1, D059, D060-1, D069, D070-5, D090, D092, D099, D370-7, D379,D380-5, D390-2, D397, D400-1, D407, D409, D410-4, D420-1, D429, D430-4, D440, D441, D443-5, D447-9, D470-5, D477, D480-7, D489, D763, R522

Appendix 2

**Table 3:** List of covariates used for calculating propensity scores. Source: [1-3] COPD: Chronic Obstructive Pulmonary Disease; eGFR: estimated Glomerular Filtration Rate; RAS: Renin-Angiotensin System; SGLT2: Sodium-Glucose Transport Protein 2; DOACs: Direct Oral Anticoagulants; NSAIDs: Non-Steroidal Anti-Inflammatory Drugs.

Basic information	Sex, age
Comorbidities	Diabetes mellitus, hypertension, dyslipidemia, myocardial infarction, stroke, heart failure, bronchial asthma, COPD, peripheral artery disease, cardiac arrest, atrial fibrillation, valve heart disease, liver disease, rheumatoid disease, psychiatry disease, bone fracture, amputation, coronary artery intervention
Laboratory data	eGFR, uric acid, hemoglobin
Medications	Aspirin, RAS inhibitors, diuretics, SGLT2 inhibitors, statins, ezetimibe, antiplatelets, DOACs, NSAIDs, calcium channel blockers, insulin, beta blockers, oral anti-diabetic agents, erythropoiesis stimulating agents, charcoal absorbents, sodium bicarbonate, potassium absorbents, phosphorus absorbents, vitamin D, iron, beta2 agonists, corticosteroid inhalers
Others	Hospital scale, department of nephrology, index date

Appendix 3 

Appendix 4 

**Table 4:** Baseline characteristics of new users of allopurinol and febuxostat after matching using propensity scores. ^1^COPD: Chronic Obstructive Pulmonary Disease; eGFR: estimated Glomerular Filtration Rate; RAS: Renin-Angiotensin System; SGLT2: Sodium-Glucose Transport Protein 2; DOACs: Direct Oral Anticoagulants; NSAIDs: Non-Steroidal Anti-Inflammatory Drugs. ^2^n (%); Median (IQR) ^3^Standardized Mean Difference ^4^CI: Confidence Interval

	Treatment		
Characteristics^1^	Allopurinol, N = 3,193^2^	Febuxostat, N = 3,193^2^	Difference^3^
Sex (men, %)	2,146 (67%)	2,201 (69%)	0.04
Age, years	78 (71, 84)	78 (71, 84)	0.03
Diabetes Mellitus, n (%)	1,105 (35%)	965 (30%)	0.09
Hypertension, n (%)	2,482 (78%)	2,469 (77%)	0.01
Dyslipidemia, n (%)	1,261 (39%)	1,216 (38%)	0.03
Myocardial infarction, n (%)	59 (1.8%)	54 (1.7%)	0.01
Stroke, n (%)	360 (11%)	376 (12%)	0.02
Heart failure, n (%)	1,261 (39%)	1,307 (41%)	0.03
Bronchial asthma, n (%)	246 (7.7%)	256 (8.0%)	0.01
COPD, n (%)	97 (3.0%)	102 (3.2%)	0.01
Peripheral artery disease, n (%)	248 (7.8%)	213 (6.7%)	0.04
Cardiac arrest, n (%)	9 (0.3%)	8 (0.3%)	0.01
Atrial fibrillation, n (%)	512 (16%)	557 (17%)	0.04
Valve heart disease, n (%)	764 (24%)	786 (25%)	0.02
Liver disease, n (%)	944 (30%)	961 (30%)	0.01
Rheumatoid disease, n (%)	203 (6.4%)	180 (5.6%)	0.03
Psychiatry disease, n (%)	128 (4.0%)	124 (3.9%)	0.01
Bone fracture, n (%)	474 (15%)	464 (15%)	0.01
Amputation, n (%)	2 (<0.1%)	3 (<0.1%)	0.01
Coronary artery intervention, n (%)	77 (2.4%)	77 (2.4%)	0.00
eGFR, ml/min/1.73m^2^	28.3 (12.9, 42.6)	27.0 (16.0, 41.3)	0.03
Uric acid, mg/dl	6.6 (5.4, 8.6)	6.0 (4.6, 7.9)	0.30
Hemoglobin, g/dl	10.7 (9.3, 12.8)	11.0 (9.6, 12.3)	0.05
Aspirin, n (%)	236 (7.4%)	220 (6.9%)	0.02
RAS inhibitors, n (%)	528 (17%)	447 (14%)	0.07
Diuretics, n (%)	691 (22%)	618 (19%)	0.06
SGLT2 inhibitors, n (%)	15 (0.5%)	14 (0.4%)	0.00
Statins, n (%)	301 (9.4%)	253 (7.9%)	0.05
Ezetimibe, n (%)	28 (0.9%)	16 (0.5%)	0.05
Antiplatelets, n (%)	307 (9.6%)	302 (9.5%)	0.01
DOACs, n (%)	113 (3.5%)	144 (4.5%)	0.05
NSAIDs, n (%)	490 (15%)	526 (16%)	0.03
Calcium channel blockers, n (%)	654 (20%)	566 (18%)	0.07
Insulin, n (%)	369 (12%)	342 (11%)	0.03
Beta blockers, n (%)	326 (10%)	326 (10%)	0.00
Oral anti-diabetic agents, n (%)	318 (10.0%)	265 (8.3%)	0.06
Erythropoiesis-stimulating agents, n (%)	420 (13%)	367 (11%)	0.05
Charcoal absorbents, n (%)	158 (4.9%)	154 (4.8%)	0.01
Sodium bicarbonate, n (%)	239 (7.5%)	178 (5.6%)	0.08
Potassium absorbents, n (%)	290 (9.1%)	259 (8.1%)	0.03
Phosphorus absorbents, n (%)	68 (2.1%)	67 (2.1%)	0.00
Vitamin D, n (%)	62 (1.9%)	59 (1.8%)	0.01
Iron, n (%)	190 (6.0%)	194 (6.1%)	0.01
Beta2 agonists, n (%)	106 (3.3%)	109 (3.4%)	0.01
Corticosteroid inhalers, n (%)	36 (1.1%)	33 (1.0%)	0.01
Hospital scale, n (%)			0.07
<200 beds	335 (10%)	353 (11%)	
200-499 beds	1,794 (56%)	1,901 (60%)	
≥500 beds	1,064 (33%)	939 (29%)	
Department (nephrology), n (%)	301 (9.4%)	167 (5.2%)	0.16
Index date, months	53 (33, 71)	52 (33, 69)	0.02
